# Hydrothermal Alteration of the Ocean Crust and Patterns in Mineralization With Depth as Measured by Micro‐Imaging Infrared Spectroscopy

**DOI:** 10.1029/2021JB021976

**Published:** 2021-08-24

**Authors:** Rebecca N. Greenberger, Michelle Harris, Bethany L. Ehlmann, Molly A. Crotteau, Peter B. Kelemen, Craig E. Manning, Damon A. H. Teagle, the Oman Drilling Project Science Team

**Affiliations:** ^1^ Division of Geological and Planetary Sciences California Institute of Technology Pasadena CA USA; ^2^ School of Geography, Earth, and Environmental Sciences Plymouth University Plymouth UK; ^3^ Department of Earth & Environmental Sciences Lamont‐Doherty Earth Observatory Columbia University Palisades NY USA; ^4^ Department of Earth, Planetary, and Space Sciences University of California Los Angeles CA USA; ^5^ School of Ocean and Earth Science National Oceanography Centre Southampton University of Southampton Southampton UK

**Keywords:** imaging spectroscopy, hyperspectral imaging, Oman drilling project, oceanic crust, hydrothermal alteration, infrared spectroscopy

## Abstract

Processes for formation, cooling, and altering Earth's ocean crust are not yet completely understood due to challenges in access and sampling. Here, we use contiguous micro‐imaging infrared spectroscopy to develop complete‐core maps of mineral occurrence and investigate spatial patterns in the hydrothermal alteration of 1.2 km of oceanic crust recovered from Oman Drilling Project Holes GT1A, GT2A, and GT3A drilled in the Samail Ophiolite, Oman. The imaging spectrometer shortwave infrared sensor measured reflectance of light at wavelengths 1.0–2.6 μm at 250–260 μm/pixel, resulting in >1 billion independent measurements. We map distributions of nine key primary and secondary minerals/mineral groups—clinopyroxene, amphibole, calcite, chlorite, epidote, gypsum, kaolinite/montmorillonite, prehnite, and zeolite—and find differences in their spatial occurrences and pervasiveness. Accuracy of spectral mapping of occurrence is 68%–100%, established using X‐ray diffraction measurements from the core description. The sheeted dikes and gabbros of upper oceanic crust Hole GT3A show more pervasive alteration and alteration dominated by chlorite, amphibole, and epidote. The foliated/layered gabbros of GT2A from intermediate crustal depths have similarly widespread chlorite but more zeolite and little amphibole and epidote. The layered gabbros of the lower oceanic crust (GT1A) have remnant pyroxene and 2X less chlorite, but alteration is extensive within and surrounding major fault zones with widespread occurrences of amphibole. The results indicate greater distribution of higher temperature alteration minerals in the upper oceanic crust relative to deeper gabbros and highlight the importance of fault zones in hydrothermal convection in the lower ocean crust.

## Key Points

- Imaging spectroscopy efficiently and effectively mapped spatial patterns in hydrothermal alteration mineral occurrence in ocean crust core
- Samail ophiolite upper ocean crust cores are dominated by chlorite, amphibole, and epidote, while deeper cores have more zeolite/prehnite
- Hydrothermal alteration largely decreases with depth in the ocean crust but is locally intense in major fault zones, even in lower crust

## 1 Introduction

The ocean crust comprises the majority of Earth's crust, yet significant questions remain in our understanding of how this basaltic and gabbroic ocean crust forms and the extent and distribution of chemical alteration from reactions with seawater. The temperature, extent, and geochemical conditions of hydrothermal alteration of the crust provide insights into mechanisms of emplacement and cooling of ocean crust and geochemical fluxes from the upper and lower crust into the ocean, which are significant in global biogeochemical cycling (e.g., Alt, 1995; Alt et al., 1986; Harris et al., 2015; Kelemen et al., 1997; Nicolas et al., 1988; Sleep, 1975). Technological and methodological challenges to complete characterization of the ocean crust are described in a review by Staudigel (2014). One challenge is the difficulty in collecting representative samples of the heterogeneous ocean crust. Access to the lower ocean crust via scientific ocean drilling holes remains difficult, and, to date, fast‐spreading lower ocean crust has only been drilled in rare tectonic windows (e.g., Gillis et al., 2014). A second means of access is collection from ophiolites, sub‐aerially exposed ancient blocks where ocean crust has been obducted onto land. Ophiolites mostly form in supra‐subduction zone settings and results might not be directly applicable to processes occurring in the major ocean basins, although the broad thermal structure is comparable. Care must be taken to separate oceanic processes from continental overprinting and surficial weathering. Consequently, outstanding questions remain about how the ocean crust formed and cooled.

Overcoming these challenges, the International Continental Scientific Drilling Program (ICDP) Oman Drilling Project (OmanDP) drilled nine boreholes in the basaltic/gabbroic ocean crust and upper mantle of the Samail Ophiolite, Oman (see Section 2) with near 100% core recovery (Kelemen et al., 2020). In addition to standard visual core description and standard geological lab techniques, the split face of the archive half of the core was scanned with an imaging spectrometer, which provides measurements indicative of mineralogy of every ∼250 × 250 μm spot in all core sections. Imaging spectroscopy of the full OmanDP core provides an unprecedented view of the mineralogy of the gabbroic/basaltic oceanic crust. This technique reduces sampling biases by objectively observing the entire core. The data set combined with the exceptional core recovery overcomes many of the challenges previously described for scientific ocean drilling expeditions, for example, the tendency of researchers to sample that which they are interested—igneous or altered rock—and traditional visual core description that relies on manual identification of minerals throughout the core with consequent variability in accuracy and consistency as personnel change and time restrictions prohibit identification of every small vein and feature (Coogan & Gillis, 2018; Staudigel, 2014).

Here, we focus on three ∼400 m long boreholes drilled into the oceanic crust, with more than 1 billion measurements of mineralogy via imaging spectroscopy: the sheeted dike‐gabbro transition, the foliated to layered gabbro transition, and layered gabbros of the lower ocean crust. Our objective is to determine the distribution of hydrothermal minerals and mineral assemblages within these ocean crust drill holes. The presence and patterns of spatial occurrence of different hydrothermal minerals and assemblages will provide objective quantification of the conditions and extent of hydrothermal exchange with depth in the ocean crust. To do this, we use the imaging spectroscopy data to determine the presence of important minerals and mineral groups at 250–260 μm spatial resolution: clinopyroxene, amphibole, chlorite, epidote, gypsum, prehnite, zeolites, kaolinite/montmorillonite, and calcite. A companion paper uses imaging spectroscopy to estimate hydration with depth (Crotteau et al., this issue). Although there are limitations of the technique, as is the case with any measurement, the accuracy of mineral identification is consistent regardless of borehole or depth, providing increased confidence in trends relative to those determined by visual logging alone. We describe the setting of the Oman ophiolite and drilling project, our methods of infrared spectroscopy, results on mineral distribution patterns with depth, and implications for the extent, mechanisms, and style of alteration of the ocean crust.

## 2 Samail Ophiolite and Oman Drilling Project

The Samail ophiolite of Oman and UAE is the best preserved and largest ophiolite in the world, with a sequence of 4–7 km of oceanic crust and 8–12 km of upper mantle peridotites (Glennie et al., 1973; Searle & Cox, 1999). The upper igneous rocks of the ocean crust formed ∼96.4–95.5 Ma, and initiation of obduction occurred within a few Myr (Rioux et al., 2012, 2013, 2016). Although most evidence points to formation in a suprasubduction zone setting prior to obduction (e.g., Lippard, 1983; MacLeod et al., 2013; Pearce et al., 1981; Searle & Cox, 1999), major and trace element concentrations show strong similarities to mid‐ocean ridge basalts (Godard et al., 2003), and seismic observations match Pacific crust (Christensen & Smewing, 1981). As such, the Samail ophiolite is widely accepted as the best on‐land analog of ocean crust and upper mantle formed at a fast spreading ridge and has consequently been the focus of geological studies of mid‐ocean processes for many decades (e.g., Gass, 1989; Pallister & Hopson, 1981).

**Figure 1:**
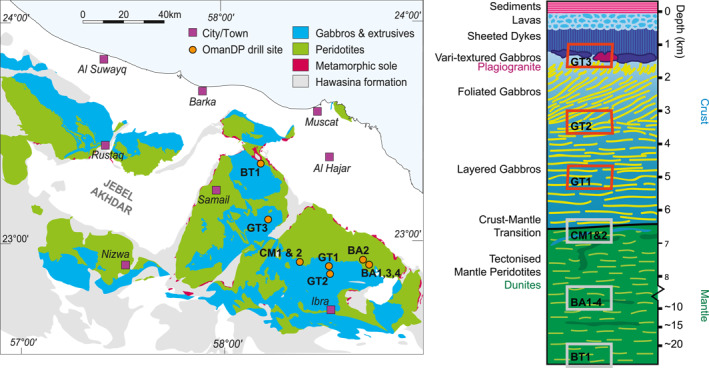
(Left) Simplified geologic map of the Samail ophiolite in Oman showing locations of boreholes drilled by OmanDP. (Right) Stratigraphy of the ophiolite with approximate positions of boreholes. Imaging spectroscopy data from Holes GT1A, GT2A, and GT3A (outlined in red) are used in this study. Modified from Kelemen et al. (2020).

The Oman Drilling Project cored 3.2 km through the ocean crust and upper mantle units of the Oman ophiolite (Figure 1; Kelemen et al., 2020). Of that, three ∼400 m deep boreholes were drilled into critical intervals of the mid to lower ocean crust (Holes GT1A, GT2A, and GT3A; Table 1), and these cores are the focus of this paper. The cores underwent the standard IODP core description and additional measurements including x‐ray computed tomography and micro‐imaging spectroscopy on the drilling vessel Chikyu, with the initial results described in Kelemen et al. (2020).

**Table 1:** Ocean Crustal Boreholes Considered in This Paper

Hole	Top depth (meters below seafloor)	Length	Description
GT3A	1500	400 m	Mid‐crust: sheeted dikes and dike‐gabbro transition
GT2A	3500	407 m	Intermediate ocean crust: foliated to layered gabbros
GT1A	5300	403 m	Lower ocean crust: layered cumulate gabbros

Borehole GT3A (Figure 1; Table 1) cored 400 m into the lowermost sheeted dike complex and the dike‐gabbro transition. The upper and lower sheeted dike lithologic units identified by the core description teams are mostly diabase and basalt. The upper gabbro unit (111.02–127.89 m depth) contains ∼54% gabbro and the lower gabbro unit (233.84–400 m) contains 45% gabbro, with the remainder being dominantly basalt and diabase. The average alteration intensity, defined by the surface area percentage of secondary minerals within an interval, estimated by visual core description, was 54%, with nearly all cores exhibiting some degree of alteration. The most common secondary minerals are albite, amphibole, epidote/clinozoisite, and chlorite (Kelemen et al., 2020, Chapter 8).

Borehole GT2A (Figure 1; Table 1) samples across the transition from foliated to layered gabbros that occur at intermediate depths in the ocean crust. Nearly all of the rocks are different types of gabbro, with olivine gabbro and olivine‐bearing gabbro accounting for 81% of the core. Visual core description estimated a mean alteration intensity of 44%, with an albite and chlorite assemblage the most common background alteration style. Albite, chlorite, and amphibole are commonly present in alteration patches and halos, and those minerals plus quartz, laumontite, prehnite, and epidote fill numerous hydrothermal veins (Kelemen et al., 2020, Chapter 7).

Borehole GT1A (Figure 1; Table 1) targeted layered cumulate gabbros of the lower ocean crust as well as a deep fault zone (Zihlmann et al., 2018). More than 87% of the core are olivine gabbro and olivine‐bearing gabbro, with other gabbros composing the remainder except for a short interval of dunite (0.3% of the total core length). Similar minerals are present as in other cores, but deformation‐related alteration is also important due to the presence of large fault zones with chlorite, amphibole, epidote, albite, and quartz background alteration and chlorite, prehnite, quartz, epidote, and clay commonly present in veins (Kelemen et al., 2020; Chapter 6).

## 3 Methods

### 3.1 Imaging Spectroscopy

Imaging spectroscopy, also known as hyperspectral imaging or spectral imaging, is a measurement technique where spatially resolved reflected light is measured at many wavelengths (Goetz et al., 1985). Vibrations of bonds within mineral structures and electronic transitions and charge transfers of transition metal cations lead to absorption features at characteristic wavelengths that fingerprint underlying mineralogy (e.g., Burns, 1993; Clark, King, et al., 1990; Hunt, 1977). The visible‐shortwave infrared (VSWIR) wavelength range measured by the instrument used here is optimal for identification of hydrated minerals, carbonates, hydrated sulfates, and many Fe^2+^‐ and Fe^3+^‐bearing minerals (e.g., Burns, 1993; Clark, King, et al., 1990; Cloutis & Gaffey, 1991; Hunt, 1977; Hunt & Ashley, 1979). However, identification of certain anhydrous minerals such as quartz, feldspars, and anhydrite is difficult to impossible in this wavelength range due to lack of diagnostic absorptions.

Laboratory imaging spectroscopy at the sample scale is rapid and non‐destructive and has numerous applications in the geological sciences (Greenberger, Mustard, Ehlmann, et al., 2015). The technique is becoming more common for measurement of drill core owing to the potential for rapid, non‐destructive determination of mineralogy (e.g., Aymerich et al., 2016; Hunt et al., 2020; Kruse et al., 2012; MacLagan et al., 2020; Mathieu et al., 2017; Speta et al., 2013, 2015) and has garnered considerable interest in the mining industry. Here, we have collected imaging spectroscopy data of all core sections recovered by OmanDP totaling 3.2 km and, for the first time, set up and used the instrument aboard the Japanese IODP drilling vessel *Chikyu*.

Methods for acquisition of the micro‐imaging spectroscopy data and full description of the instrument are described in detail in Kelemen et al. (2020) and summarized here. Micro‐imaging spectroscopy measurements were acquired on the split face of the archive half of all OmanDP core sections onboard the Chikyu. All data used in this study and scans of Holes GT1A, GT2A, and GT3A (Table 1), were collected during the ChikyuOman2017 core description campaign (July–September 2017). Measurements of up to ∼70 m of core were obtained during each 12 h shift on the Chikyu, limited in part by the rate at which the hard drive on the instrument filled, at which point data needed to be copied onto external hard drives before additional measurements could proceed.

The Caltech imaging spectrometer system, custom‐built by Headwall Photonics, Inc., has co‐boresighted visible‐near infrared (VNIR; 0.4–1.0 μm, 5 nm spectral resolution, 1.625 nm spectral sampling) and shortwave infrared (SWIR; 1.0–2.6 μm, 6 nm spectral resolution and sampling) sensors. It is a pushbroom scanner that acquires data for one spatial line at a time. As the core is moved below the instrument by a translation stage or track, the image cube builds line‐by‐line. The instrument was mounted vertically on a structure above a Geotek Multi‐Sensor Core Logger track on the Chikyu with acquisition frame periods matched to the speed of the track, and the core was illuminated with a halogen slit lamp. Spatial resolutions achieved are ∼83 μm/pixel (VNIR) and 250 μm/pixel (SWIR) for HQ‐size core (63.5 mm diameter) and 87 μm/pixel (VNIR) and 260 μm/pixel (SWIR) for NQ‐size core (47.6 mm), with differences due to the added height of the split core face for HQ core diameters bringing the surface ∼8 mm closer to the sensor. During the OmanDP drilling, HQ core was obtained from the surface to some depth, below which NQ core was drilled.

Between every ∼4 images, dark current measurements were acquired with the lens cap on, and images of a 99% Labsphere Spectralon target were taken at approximately the same height as the split core surface. These measurements were used to calibrate the data pixel‐by‐pixel to reflectance, and spectra were corrected for the absolute reflectance properties of Spectralon (*R*_*s*_):

$$R=\;\frac{S_{t}-S_{d}}{S_{s}-S_{d}}*R_{s},$$

where *R* is the reflectance, *S*_*t*_ is the signal received from the measured target, *S*_*d*_ is the dark current measurement, and *S*_*s*_ is the signal received from the Spectralon measurement. In addition, a calibration target with 8 panels of different reflectance values was placed in front of every core section that was scanned to provide additional points of validation in each image.

Analysis of the imaging spectroscopy data was automated, was done on each pixel, and primarily occurred through calculation of spectral parameters (Clark & Roush, 1984; Pelkey et al., 2007; Viviano‐Beck et al., 2014) for the presence or absence of key absorption features and then using those parameters to develop mineral indicators, similar to the workflow of Greenberger et al. (2020. This approach was selected over pattern matching algorithms such as 216 spectral feature fitting (Clark, Gallagher, & Swayzel, 1990) or Tetracorder (Clark et al., 2003), which require spectral libraries containing every mineral within the image, including every solid solution composition. The large data volume combined with high spatial resolution of our data set permits analysis of individual grains. The complete data set contains solid solution compositions that are not encompassed by existing spectral libraries (Kokaly et al., 2017; Murchie et al., 2007), though we can still recognize mineral classes and most solid solution variations because shifts in the wavelength position of absorption features have been characterized in the literature. Formulas for calculation of spectral parameters are given in Table A1. Formulas for the following step, aggregation of parameters to mineral indicators, are given in Table A2, following similar workflows to Greenberger et al. (2015, 2020). These provide mineral identifications and quantitative parameterizations of mineral occurrence, but occurrence% should not be construed at this point as quantitative indication of wt% or vol%. Mineral indicators were smoothed with a 3 × 3 median filter to reduce noise.

Thousands of images totaling terabytes of data were analyzed in this study, using images of all core sections from Holes GT1A, GT2A, and GT3A, other than those designated “M” for miscellaneous due to overlap with core previously drilled. As a result, automated methods were used to analyze and aggregate data. Within images of the core, other materials were present, including a calibration target, the edge and body of the plastic core liner, Styrofoam, and shadows or dark space. Eliminating the first 300 lines from each image typically removed the calibration target, and we therefore removed these lines before further analysis. Then, parameters were calculated to identify other non‐rock materials, including BD1200, BD1715, and BD2106 (Table A1), and thresholds given in Table A2 generally mask these materials well. Dark pixels in shadows where there are fractures within the rock and outside of the core liner on the edges of the image were identified through iterative testing and masked using reflectance values <0.02 at wavelength 1.11 μm or values both <0.035 at 1.11 μm and <0.02 at 2.41 μm.

For single image analysis, the first 300 lines and these non‐rock materials were eliminated. To determine downhole trends on a constant depth scale, depths were determined for each line within the image. The top of each core section was identified as the first 20 consecutive lines (∼5 mm vertically) with at least 50 pixels horizontally (12.5 mm) that contained rock (i.e., none of the materials described above), and the bottom was determined to be the last 20 lines with at least 50 rock pixels horizontally. Depths for each imaging spectroscopy line within the core were extrapolated using recorded depths of each core section. Minor errors in depth up to a few 10's of cm occur when the beginning or end of the core is void space and filled with Styrofoam, which the mask identifies as non‐rock material. Because each core section is up to ∼1 m in length, these errors move the 1 m bin in which statistics are aggregated by no more than 1 m. Once established, following iterative optimization and manual examination of a representative subset, data processing times were on the order of minutes per image to do the initial calibration to reflectance and mineral mapping, and aggregation of downhole data took less than one day per borehole, though we note that further optimization of code and/or differences in computing resources would affect the processing times.

Hence, we report the %occurrence. Determination of quantitative mineral abundances (%wt. or %vol) is difficult with shortwave infrared spectroscopy of simple, controlled particulate mixtures (see methods and accuracy of Hapke, 1981; Lapotre et al., 2017; Mustard & Pieters, 1989; Shkuratov et al., 1999) and beyond the present scope of this work for the initial analysis of these complex rocks. Use of metrics such as % occurrence, the number of pixels containing minerals of interest, provides important constraints on the extent of hydrothermal circulation and identifies minerals present in lower abundances due to partial retrograde metamorphism or overprinting. Spectra from a representative subset of cores were examined manually to validate the results of automated mapping and understand the range of spectral signatures present; the downhole mineral abundance changes were determined by applying the automated methods.

### 3.2 Validation Data Sets

For validation of mineral identifications via spectroscopy, we compare the spectral interpretations with X‐ray diffraction (XRD) measurements and thin section petrography. All measurements and sample preparation were performed by the Oman Drilling Project core description teams aboard the *Chikyu* following methods described in and results reported by Kelemen et al. (2020). These XRD measurements were obtained on small veins and intervals of rock. We match sampling locations to locations within the images and compare the mineralogy. For petrographic analyses, the billets that remained after thin section preparation were scanned with the imaging spectrometer with the same methods as imaging of the full core. These samples pair with thin sections analyzed through traditional petrography to further determine the accuracy of the imaging.

We conducted a detailed analysis using the depth of the XRD measurements to estimate the location of the sampling, manually checking for the presence of each mineral identified with XRD in the imaging spectroscopy data and allowing a small radius on the order of mm's surrounding the sampling location. For example, when veins were sampled for XRD measurements, we checked for the presence of XRD mineral identifications in imaging spectroscopy mapping of the vein and the matrix immediately surrounding the vein; it is quite plausible for small veins that the surrounding matrix was sampled as well. We do note as a possible source of error that there may be slight differences between the working half of the core and the archive half in terms of what is present at each precise depth. All samples used for comparison and depths within each core section are given in Table S2, and the full mineral occurrence mappings are available in Files S2–S4.

## 4 Results

### 4.1 Infrared Spectral Signatures

**Figure 2:**
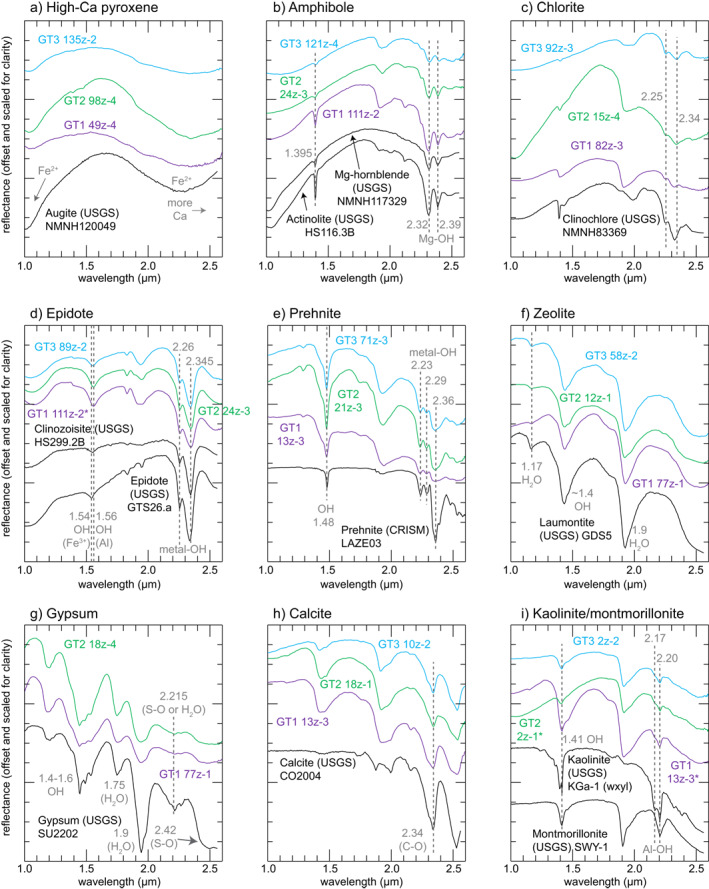
Spectra of pixels with spectral signatures dominated by minerals mapped in this study and corresponding laboratory spectra from the United States Geological Survey (USGS; Kokaly et al., 2017) and Compact Reconnaissance Imaging Spectrometer for Mars (CRISM; Murchie et al., 2007) spectral libraries. Variations between spectra from images and library spectra are due to subpixel mixing with other minerals in the rocks in this study. Where possible, spectra were obtained from core sections where these minerals were identified via X‐ray diffraction (XRD) within 10’s of cm. Asterisk (*) indicates spectra where XRD samples were not available. Dashed lines show positions of key absorption features. All spectra are 5 × 5 pixel averages. Coordinates of pixels where spectra were obtained are given in Table S1.

Pixels containing pyroxene (Figure 2a) have broad electronic transitions due to Fe^2+^ in the M2 site at wavelengths ∼2.0 and ∼1.0 μm (Adams, 1974; Burns, 1993; Cloutis & Gaffey, 1991). The wavelength minimum of the longer wavelength absorption feature at >2.20 μm indicates that most pyroxenes identified spectrally in these cores are high‐Ca pyroxenes, that is, pyroxenes in the upper portion of the pyroxene quadrilateral, including clinopyroxenes augite and diopside but not pigeonite (Cloutis & Gaffey, 1991; Klima et al., 2011). These clinopyroxenes are most likely magmatic, though we cannot rule out that there may be occasional secondary pyroxenes. Spectra of pyroxenes within this core frequently also contain weak, narrow vibrational absorption features at 1.39–1.57 μm (OH overtone), 1.9 μm (H‐O‐H combination), and 2.2–2.4 μm (metal‐OH combinations) (e.g., Clark, King, et al., 1990), suggesting minor alteration and hydration of the pyroxenes.

Amphibole spectra (Figure 2b) are characterized by an OH overtone at 1.395 μm and Fe/Mg‐OH combination bands at 2.25, 2.31–2.32 μm and 2.39–2.40 μm (Laukamp et al., 2012; Mustard, 1992). Although the 2.31–2.32 μm absorption feature overlaps with other minerals, the ∼2.39 μm feature is only present in a few minerals, the others being talc and saponite, which are rare in the spectroscopy data set. Amphibole spectra in these cores typically have a 2.12 μm feature, which helps distinguish them from talc (Laukamp et al., 2012), though mixing and low abundances of amphibole may prevent detection of this feature.

Chlorite (Figure 2c) is identified by the presence of the OH overtone at ∼1.39 μm and metal‐OH combination absorptions at 2.25 μm and principal absorption centered from 2.33 to 2.35 μm, with position dependent on Mg/Fe content (e.g., Bishop et al., 2008; Clark, King, et al., 1990). The absence of an absorption feature at 2.39 μm distinguishes pixels containing chlorite from amphibole (e.g., Laukamp et al., 2012). Although chlorite and amphibole commonly occur together in the core, chlorite is difficult to identify where amphibole is present because the absorption features in chlorite also are present in amphibole (which has the additional 2.39 μm feature), and we therefore do not map chlorite in pixels where amphibole is identified.

The diagnostic absorption feature of epidote‐clinozoisite (Figure 2d) is an OH stretching overtone at ∼1.55 μm that systematically shifts to longer wavelengths with increasing Al content relative to Fe^3+^, with the longest wavelengths indicating clinozoisite compositions (White et al., 2017). Epidote also has metal‐OH absorption features at 2.26 and 2.35 μm.

Prehnite (Figure 2e) is identified by the presence of the OH overtone at 1.48 μm (Clark, Gallagher, & Swayze, 1990; White et al., 2017). There are also shallow metal‐OH combination absorptions at 2.23 and 2.29 μm followed by a deeper absorption at 2.36 μm.

Zeolite spectra (Figure 2f) are characterized by deep absorption features due to OH and H_2_O. The H‐O‐H combination absorption occurs at 1.9 μm and also ∼1.17 μm, and the OH overtone is observed at 1.4 μm (Cloutis et al., 2002). Zeolites such as laumontite typically have a shoulder at 1.75–1.80 μm, as can be seen in Figure 2f. Analcime has a deeper absorption at 1.79 μm, permitting its discrimination from laumontite (Kokaly et al., 2017), and thomsonite has a more complex series of absorption features in both the 1.4 and 1.9 μm region due to clusters of H_2_O around cations within its structure as well as potentially multiple cations, Ca and Na (Cloutis et al., 2002). Because OH and H_2_O are present in many minerals, it can be difficult to identify zeolites where other hydrated minerals are present, as the spectral features due to hydration overlap. Therefore, we do not map zeolite if strong metal‐OH features are observed.

Gypsum (Figure 2g) has a characteristic triplet at 1.4–1.6 μm due to H_2_O overtones and combinations and a deep 1.9 μm H_2_O absorption (Clark, King, et al., 1990; Cloutis et al., 2006; Hunt et al., 1971). A diagnostic spectral feature with little overlap with other minerals here is an absorption feature at 1.75 μm due to H_2_O (Cloutis et al., 2006). Gypsum spectra also have a weaker absorption feature at ∼2.21 μm due to S‐O or H_2_O and a shoulder at ∼2.42 μm from S‐O (Clark, King, et al., 1990; Cloutis et al., 2006; Hunt et al., 1971).

Absorption features in calcite spectra (Figure 2h) occur at 2.34 μm (third C‐O asymmetric stretching overtone) and 2.5 μm (C‐O combination) (Gaffey, 1985, 1986, 1987; Hunt & Salisbury, 1971). Calcite can be distinguished from other carbonate minerals by the wavelength of the minimum at 2.29–2.35 μm and occurs at 2.34 μm. Identifying calcite in mixtures with chlorite, epidote, prehnite, and amphibole is a known problem, as the 2.34 μm feature in calcite overlaps with other minerals (Dalton et al., 2004). Dalton et al. (2004) used laboratory mixtures of chlorite, epidote, and calcite to improve detection and abundance quantification but found that the minerals in the mixture must precisely match the solid solution compositions of the minerals in the target, a challenge in 1.2 km of core. We therefore do not map calcite when chlorite, epidote, amphibole, or prehnite are present. However, the high spatial resolution of these measurements does permit identification of calcite veins when subpixel mixing does not occur.

Kaolinite spectra (Figure 2i) exhibit doublets at ∼1.4 μm due to OH and ∼2.17/2.20 μm due to an Al‐OH combination (e.g., Bishop et al., 2008; Clark, King, et al., 1990). Montmorillonite spectra have an OH overtone at 1.41 μm, an H_2_O combination band at ∼1.9 μm and an Al‐OH combination at 2.21 μm, and, unlike kaolinite, the 1.41 and 2.21 μm features are not doublets (Bishop et al., 2008). While the doublets are moderately defined in spectra shown in Figure 2i, they are only sometimes observed in spectra of these cores, suggesting the presence of both kaolinite and montmorillonite. Where poorly or moderately defined doublets are present, the kaolinite is likely mixed with montmorillonite or other minerals or there are differences in crystallinity with library spectra.

**Figure 3:**
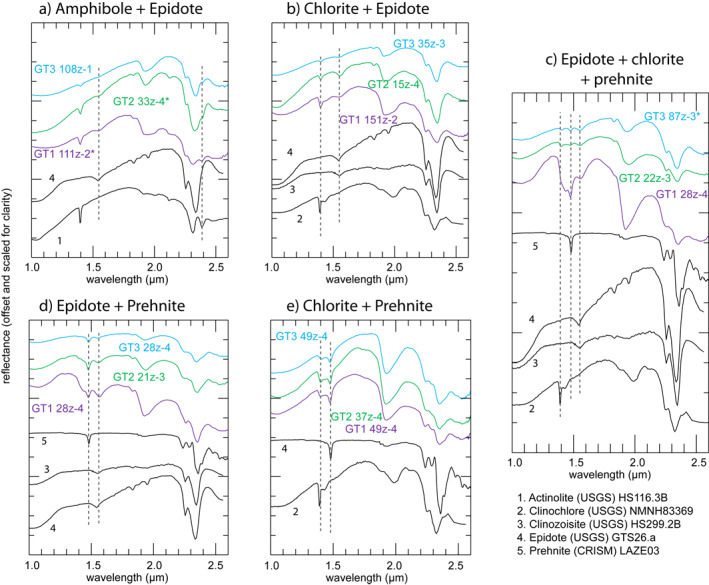
Spectra of pixels with multiple intergrown minerals and corresponding laboratory spectra from the United States Geological Survey (Kokaly et al., 2017) and Compact Reconnaissance Imaging Spectrometer for Mars (Murchie et al., 2007) spectral libraries. Variations between spectra from images and library spectra are due to subpixel mixing with other minerals in the rocks in this study. Where possible, spectra were obtained from core sections where these minerals were identified via X‐ray diffraction (XRD) within 10’s of cm. Asterisk (*) indicates spectra where XRD samples were not available. Dashed lines show positions of key absorption features. Coordinates of pixels where spectra were obtained are given in Table S1.

Certain mixtures of minerals are mapped at the subpixel scale (Figure 3), and key absorption features are used to identify minerals present within these mixtures. An absorption feature at 1.54–1.57 μm indicates that epidote is present (Figures 3a–3d). A 2.39 μm feature suggests amphibole (Figure 3a). The 1.48 μm feature, where gypsum is not present, signifies the presence of prehnite (Figures 3c–3e). Chlorite is inferred to be present if there are 1.39 μm and 2.32–2.35 μm features (Figures 3b, 3c and 3e) but no 2.39 μm absorption, though there is potential for misidentification of chlorite where other Fe/Mg‐OH‐bearing minerals such as serpentine occur (Fe/Mg‐bearing smectites other than the saponite endmember tend to have shorter wavelength 2.29–2.31 μm absorptions). Other assemblages of minerals mixed at the sub‐pixel scale also occur in these cores that are not shown in Figure 3 or in color maps (e.g., Text S2–S4), such as prehnite (1.48 μm feature present) and amphibole (2.39 μm feature); the identification is still recorded and used in the results of this paper but not shown in color maps for graphical simplicity.

Additional minerals are known to occur in these rocks (Kelemen et al., 2020) but are not mapped. Quartz and albite are the most common, and these are transparent at VSWIR wavelengths. The same is true of anhydrite and other feldspars. While these minerals may raise the overall albedo of the rock, the lack of diagnostic absorption features in this wavelength range makes them difficult to impossible to map here. A feature due to Si‐OH at ∼2.2 μm can be seen in spectra of quartz when it has trace water (e.g., Aines & Rossman, 1984) but is quickly overwhelmed by low abundances of other minerals in the same pixel. Similarly, pure, unaltered plagioclase with trace amounts of Fe has a weak, broad absorption feature at ∼1.25 μm but is only typically seen at concentrations >90% (Cheek & Pieters, 2014). We note that this may limit applicability of the SWIR spectroscopy in studies of the igneous rocks in more plagioclase‐rich portions of the crust, if sufficient alteration or pyroxene are present to overwhelm the plagioclase spectral signature. Fine‐grained disseminated oxide and sulfide minerals serve to darken the rock and reduce the intensity of absorption features due to other minerals (Morris et al., 1985), but they typically lack their own diagnostic absorption features and are not mapped. Serpentines are occasionally present, but, since textural and subpixel mixing relationships generally obscure the 2.1 μm absorption within these rocks (King & Clark, 1989), they are difficult to distinguish from chlorite and may co‐occur with chlorite. Other clay minerals such as saponite and vermiculite were identified via XRD but are not abundant enough in the cores to contribute to the overall trends discussed in this paper. While orthopyroxene and pigeonite may be present, and limited intervals of orthopyroxene‐bearing gabbros were identified in GT1A and GT2A (Kelemen et al., 2020), pyroxenes identified through XRD were nearly always augite or diopside, and we only map these pyroxenes with higher Ca content in this paper.

### 4.2 Validation and Comparisons With Thin Section Petrography and X‐Ray Diffraction

**Figure 4:**
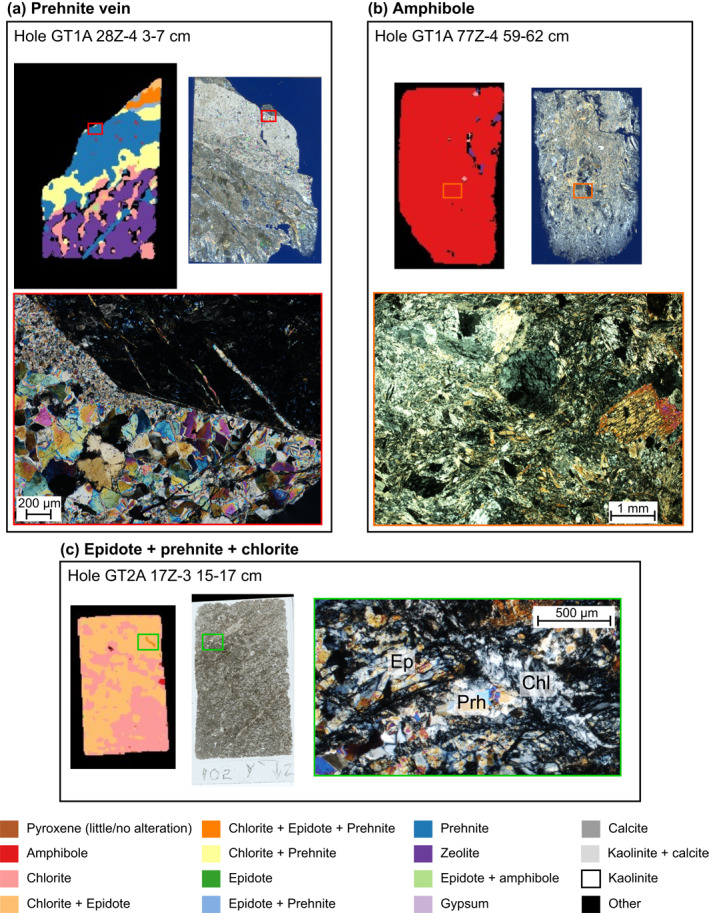
Examples of thin section validation of imaging spectroscopy. (a) GT1A 28Z‐4 3–7 cm, red box highlights the area of photomicrograph (below) on mineral map from imaging spectroscopy (left) and whole thin section scan (right). A large cm scale prehnite vein dominates this sample, confirmed by both thin section and IR. (b) GT1A 77Z‐4 59–62 cm orange box highlights the area of photomicrograph (below) on spectral map dominated by amphibole (left) and whole thin section scan (right). This sample is part of a fault zone with extensive replacement to amphibole, and amphibole is clearly seen as both larger crystals and finer grained groundmass in this fault zone. (c) GT2A 17Z‐3 15–17 cm green box highlights the area of photomicrograph (right) on imaging spectroscopy map (left) and whole thin section scan (middle). This area hosts a mixed assemblage of fine grained chlorite + epidote + prehnite and the spectroscopy map picks this assemblage out successfully. Some resolution is lost in the cross cutting vein relationships.

**Figure 5:**
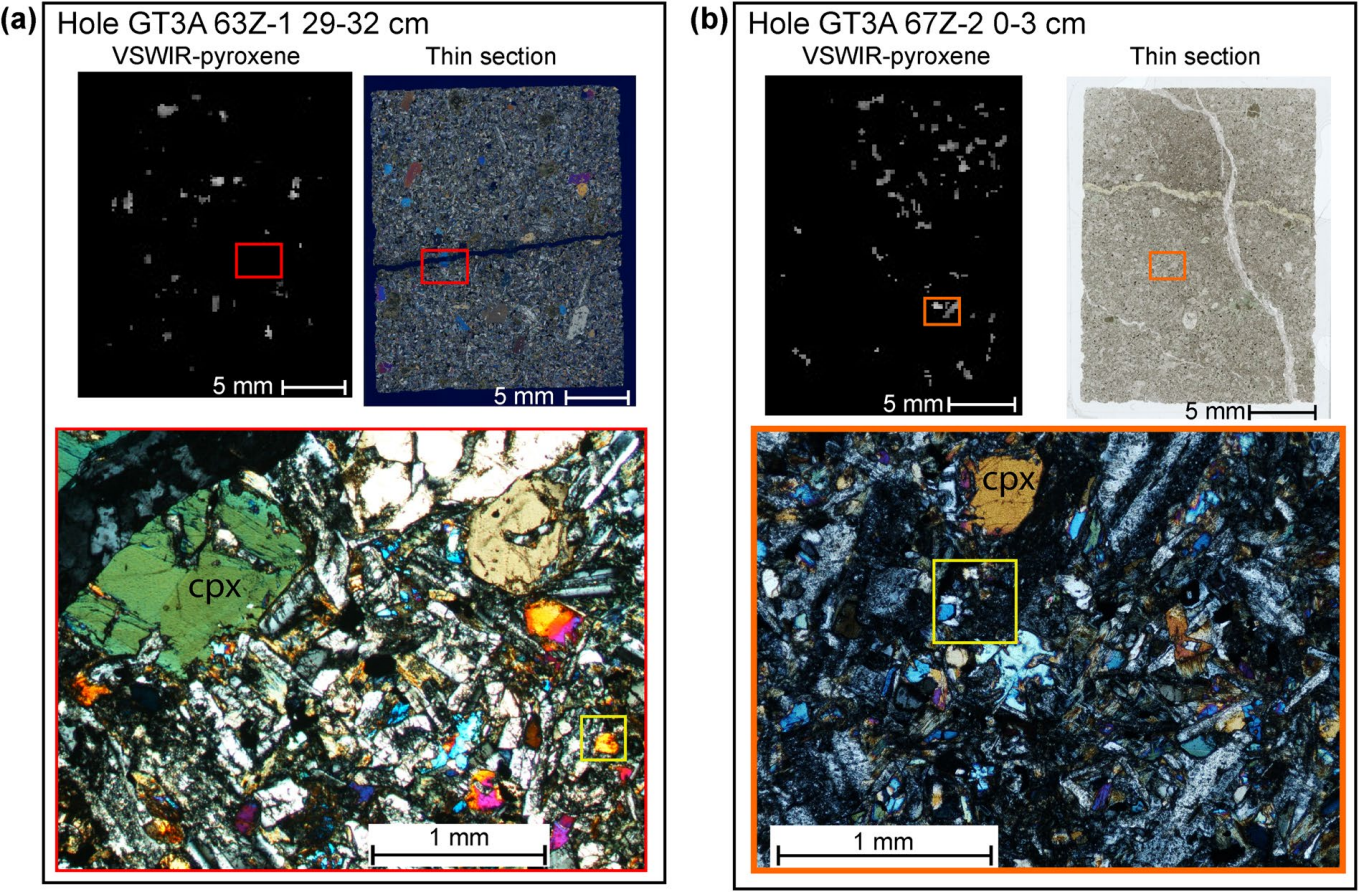
Comparison of clinopyroxene occurrence maps from imaging spectroscopy (left; grayscale—brighter represents deeper pyroxene absorption features and black is no pyroxene) and thin section petrography for the identification of clinopyroxene. In thin section both samples (a) GT3A 63Z‐1 29–32 cm and (b) GT3A 67Z‐2 0–3 cm show some fresh clinopyroxene at grain sizes from <100 microns up to 1 mm. The yellow boxes represent the size of an individual pixel in the spectroscopy data set and enclose a fresh clinopyroxene. The finer grained clinopyroxene is less evident in the imaging spectroscopy, likely due to alteration in the surrounding matrix and/or the 3 × 3 median filter that was applied.

Thin section petrography confirms that the minerals identified by imaging spectroscopy exist within the OmanDP cores. Imaging spectroscopy measurements of the thin section billets and petrography of the accompanying thin sections permits comparison at the qualitative level. Areas of single mineral occurrence (Figures 4a and 4b) and mixed mineral assemblage (Figure 4c) identified with imaging spectroscopy of the billets were confirmed in thin section. Thin section petrography highlights some limitations of the imaging spectroscopy data set. Grain sizes for secondary minerals, and in some intervals the primary minerals, can be <100 μm in size. The imaging spectroscopy data for these scans has a resolution of 250–260 μm, which means that if a grain is below this size or straddles two pixels, it may not be identified, particularly for clinopyroxene and minerals such as calcite that are automatically excluded by the analysis algorithm where minerals with overlapping absorption features are present (see Absent column of Table A2). At a qualitative level, there is evidence that the imaging spectroscopy data set underestimates the presence of clinopyroxenes when they occur at small crystal sizes (Figure 5). However, thin section petrography confirms the relative trends in abundance. Any under or overestimations of mineral occurrence will impact each borehole in the same way and relative changes within each hole are considered representative and consistent with thin section observations.

A common feature of the hydrothermal alteration of the OmanDP cores is incipient alteration that records the initial stages of replacement and is characterized by very fine scale inclusions of secondary minerals where the crystals otherwise appear relatively “fresh,” for example, the breakdown of clinopyroxene to amphiboles. Comparison of thin sections and the imaging spectroscopy of the thin section billets suggests spectroscopy at these wavelengths is very sensitive to the incipient alteration, either due to non‐linearities in the spectral mixing between the components (e.g., Hapke, 1981), or sufficient distortion of the pyroxene structure, where a clinopyroxene can look “fresh” petrographically but in fact is mapped as predominantly amphibole or chlorite. Therefore, clinopyroxene spatial occurrences may be underestimated while petrographically minor hydrated minerals are well‐detected, contributing to the overall spatial occurrence percentages of secondary minerals. These examples demonstrate the necessity to have a good understanding of the textures of secondary minerals when interpreting the imaging spectroscopy data sets.

**Figure 6:**
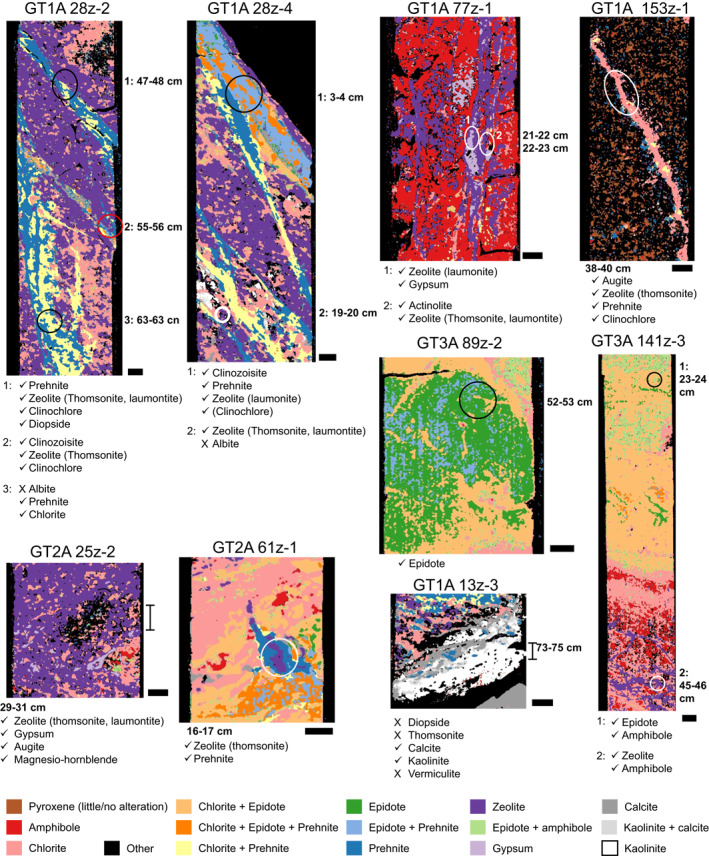
Mineral maps derived from imaging spectroscopy with approximate locations of X‐ray diffraction (XRD) samples from the OmanDP core description (Kelemen et al., 2020). XRD identifications are listed below each mineral map. Check marks indicate minerals identified by both XRD and imaging spectroscopy, and X's indicate minerals identified by XRD but not imaging spectroscopy. Particular amphibole mineral identifications are given where available, though some are only listed as amphibole in the core description (Kelemen et al., 2020). Note that there are minor differences in the measurements, as imaging spectroscopy measurements are of the split face of the archive half of the core, while material was sampled for XRD from the paired face of the working half of the core. Scale bars are 1 cm.

We use XRD measurements from the OmanDP core description (Kelemen et al., 2020) to quantitatively assess the accuracy of the imaging spectroscopy mineral mapping and find accuracies of >80% in identification of minerals detected with XRD other than calcite and kaolinite/montmorillonite (Table 2 and S2). Here, we define the accuracy for each mineral group as the rate at which imaging spectroscopy positively identifies minerals that were detected via XRD; this is the true positive rate. Examples of mineral maps and XRD results are shown in Figure 6. Minerals with the lowest accuracy are pyroxene (82%), gypsum (85%), and calcite (69%). The lower accuracy of gypsum identifications may be due to the small number of XRD measurements that included this mineral, as it was only missed in the spectroscopic mineral mapping two times out of 13. Calcite detections are limited by the difficulty of identifying it when intergrown with chlorite, epidote, amphibole, and prehnite (see Section 4.1 and notes in Table 2). For pyroxene, as suggested in the thin section data, signatures of alteration minerals may overwhelm the spectral signature of pyroxene, despite residual pyroxene being present (e.g., Leask & Ehlmann, 2016), and the thresholds in our algorithm prohibit identification of pyroxene in pixels with strong hydration signatures (Table A2). The rates of detection of amphibole, chlorite, and epidote/clinozoisite are all 100%, and prehnite and zeolite are also >90%. Missed instances of zeolite may result from the presence of other hydrated minerals at subpixel spatial resolution. As discussed in Section 4.1, it is difficult to identify zeolite, which has a spectral signature dominated by H_2_O, where other hydrated minerals with H_2_O occur. Other minerals such as quartz, albite, and anhydrite are identified through XRD but are not mapped in this paper. There are too few XRD detections of kaolinite/montmorillonite to assess our accuracy rigorously.

**Table 2:** Accuracy in Spectral Identification of Minerals Determined by XRD to Be Present

Mineral group	Identified	Not identified	Accuracy	Notes
Pyroxene	62	14	82%	Pyroxene is not identified in pixels that also contain strong spectral evidence for hydrated minerals
Amphibole	77	0	100%	
Chlorite	111	0	100%	Chlorite is not mapped in pixels where amphibole is identified.
Epidote	34	0	100%	Includes clinozoisite
Prehnite	95	2	98%	
Zeolite	117	8	94%	Zeolite spectra are dominated by hydration features, which are present (but weaker and sometimes narrower) in other hydrated minerals. Zeolites are not identified in pixels with chlorite, epidote, or prehnite.
Gypsum	11	2	85%	
Calcite	11	5	69%	The main spectral feature of calcite in this wavelength range at 2.34 μm overlaps with a metal‐OH feature in chlorite, epidote, prehnite, and amphibole (Dalton et al., 2004). Calcite is not mapped in pixels where these other minerals are identified.

*Note*. Kaolinite/montmorillonite are not included in this table because there were too few identifications with XRD to obtain useful statistics.

Abbreviation: XRD, X‐ray diffraction.

Although, we can determine accuracy or true positive rate (identified in both imaging spectroscopy and XRD; “identified” column of Table 2) and false negative rate (identified in XRD but not imaging spectroscopy; “not identified” column of Table 2), it is difficult to determine the rate of false positives (identified with imaging spectroscopy/not identified with XRD) and true negatives (not identified with either technique) because the width of the collected core sample is unknown, and samples were obtained from the working half of the split core, not the archive half that was imaged and separated by the thickness of the saw blade. It is impossible to determine whether, for example, a small amount of material from outside of a vein was collected during sampling or whether our identification of that material is a false positive. Nevertheless, there are a few cases where XRD identified minerals such as prehnite in a sample and did not identify chlorite, but we map every pixel containing prehnite as also having chlorite. Therefore, chlorite is likely slightly overestimated. Most often, serpentine and, less frequently, low abundances of amphibole particularly in mixtures with epidote have been mistaken in our algorithm for chlorite. We do not see obvious indications of false positives for other minerals with XRD.

### 4.3 Mapping of Ocean Crust Cores

**Figure 7:**
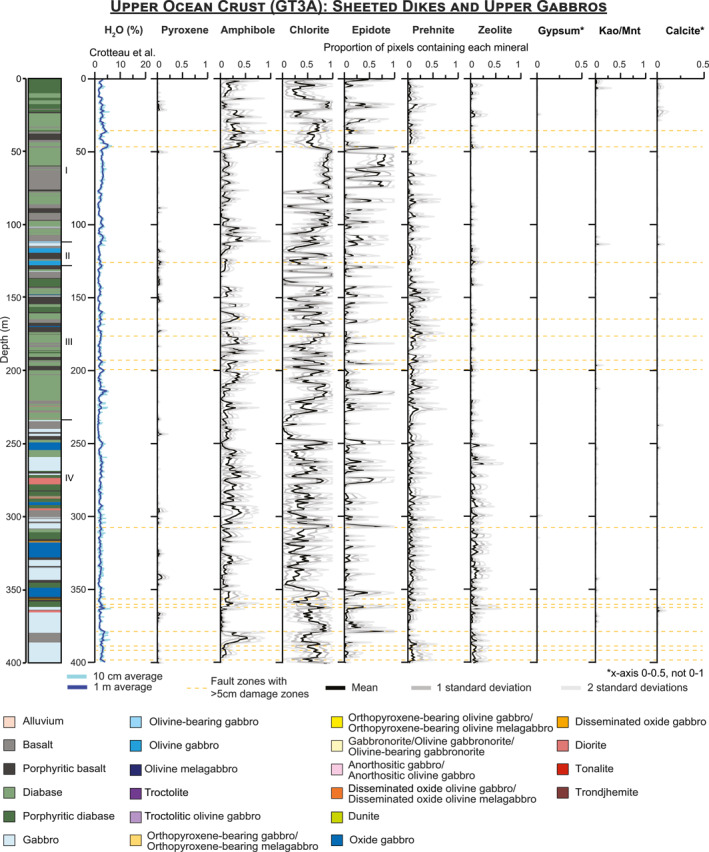
Downhole plots for the sheeted dikes and upper gabbros of Hole GT3A showing the H_2_O content with 10 cm and 1 m averaging from Crotteau et al. (this issue) and occurrences of minerals mapped from imaging spectroscopy data. Mineral occurrences are calculated using the percentage of pixels interpreted to contain the mineral in every 250–260 μm line in the paper, with averages and standard deviations calculated on the line by line percentages within each 1 m increment. The stratigraphy on the left and locations of fault zones are from Kelemen et al. (2020). Kln/Mnt = kaolinite/montmorillonite.

**Figure 8:**
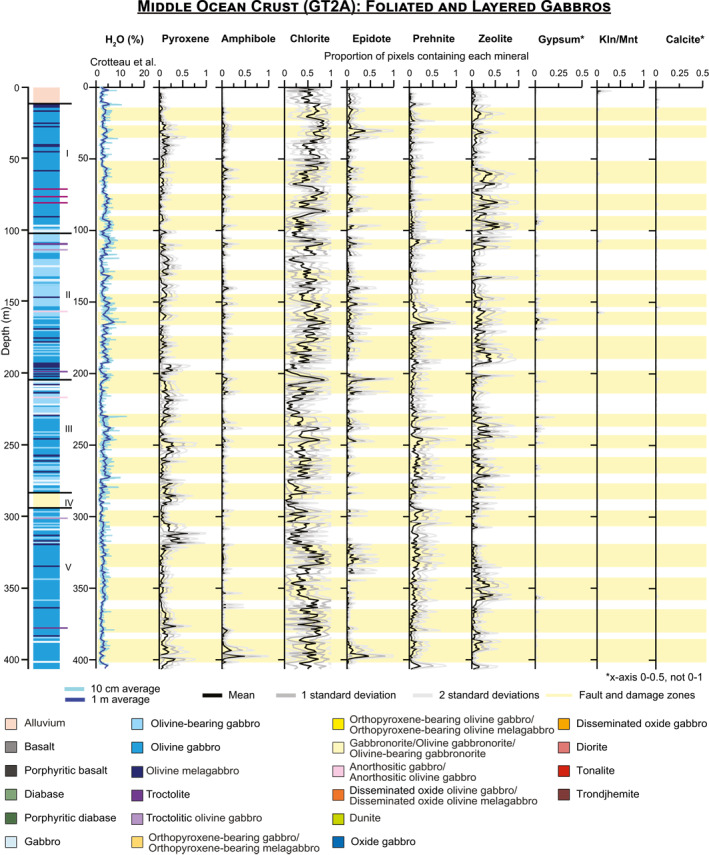
Downhole plots for the foliated and layered gabbros of Hole GT2A showing the H_2_O content with 10 cm and 1 m averaging from Crotteau et al. (this issue) and occurrences of minerals mapped from imaging spectroscopy data. Mineral occurrences are calculated using the percentage of pixels interpreted to contain the mineral in every 250–260 μm line in the study, with averages and standard deviations calculated on the line by line percentages within each 1‐m increment. The stratigraphy on the left and fault and damage zones are from Kelemen et al. (2020). Kln/Mnt = kaolinite/montmorillonite.

**Figure 9:**
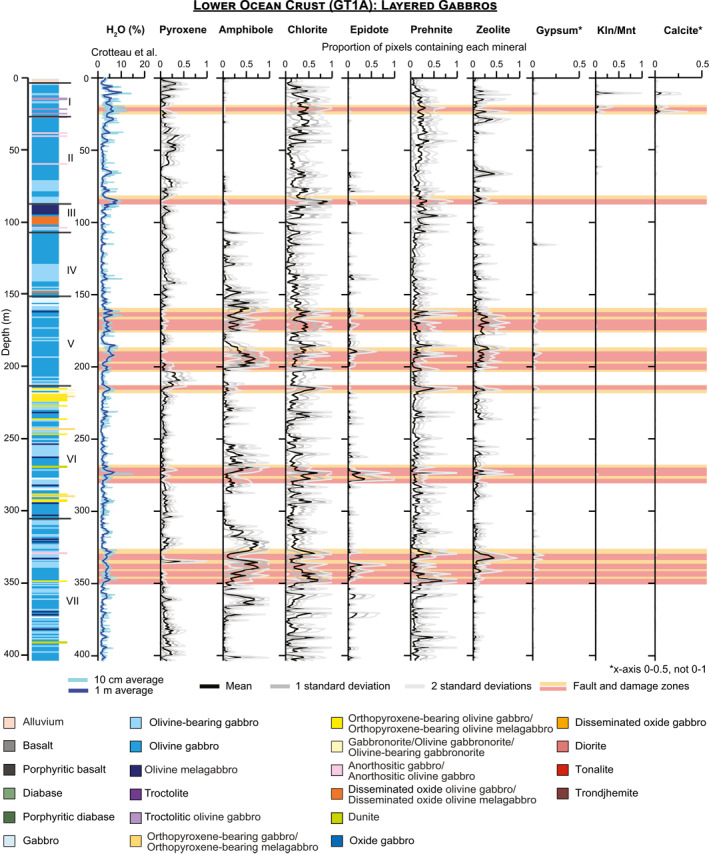
Downhole plots for the layered gabbros of Hole GT1A showing the H_2_O content with 10 cm and 1 m averaging from Crotteau et al. (this issue) and occurrences of minerals mapped from imaging spectroscopy data. Mineral occurrences are calculated using the percentage of pixels interpreted to contain the mineral in every 250–260 μm line in the study, with averages and standard deviations calculated on the line by line percentages within each 1 m increment. The stratigraphy on the left and locations of major fault and damage zones are from Kelemen et al. (2020). Kln/Mnt = kaolinite/montmorillonite.

The algorithms used to map minerals were applied to every image of every core section (Files S2–S4), and standard downhole plots showing the proportion of pixels containing each mineral with depth are presented for OmanDP Holes GT3A (Figure 7), GT2A (Figure 8), and GT1A (Figure 9). We also calculate the percentage of pixels in each core interval in which each mineral is identified (Table 3), that is, % occurrence (see Section 3.1).

**Table 3:** Percentage of Holes GT3A (Dike‐Gabbro Transition), GT2A (Foliated to Layered Gabbros), and GT1A (Layered Gabbros/Major Fault Zones) Containing Each Mineral (%Occurrence)

Hole	Cpx	Amp	Chl	Ep/Czo	Prh	Zeo	Gp	Cal	Kln‐Mnt	Unclassified
GT3A	0.8%	15%	51%	12%	6%	3%	0.02%	2%	0.1%	28%
GT2A	10%	3%	46%	4%	8%	12%	0.1%	2%	0.1%	29%
GT1A	9%	13%	22%	2%	14%	6%	0.1%	2%	0.3%	44%

*Note*. Because most pixels contain more than one mineral, the numbers for each borehole do not sum to 100%. Incipiently altered pyroxene, when present, is likely often classified as an alteration phase; see Section 4.3. Percentages reported are weighted to remove biases for NQ core due to its reduced spatial resolution and width.

Abbreviations: Amp, amphibole; Cal, calcite; Chl, chlorite; Cpx, clinopyroxene; Ep/Czo, epidote/clinozoisite; Gp, gypsum; Prh, prehnite; Zeo, zeolite; Kln‐Mnt, kaolinite/montmorillonite (Whitney & Evans, 2010).

Pyroxene, amphibole, chlorite, epidote, prehnite, zeolite, gypsum, carbonate, and kaolinite/montmorillonite are identified in every borehole but in different proportions (Table 3), though gypsum is exceedingly rare in GT3A. We also track the number of pixels where none of these minerals are identified, which range from 28% in GT3A to 44% in GT1A. These pixels contain plagioclase, quartz, or other minerals that are not mapped, or they may contain fine‐grained oxides or sulfides that darken and obscure the spectral signatures of other minerals present.

## 5 Discussion

### 5.1 General Trends in Hydration and Mineralogy

Consistent with the OmanDP core description (Kelemen et al., 2020), the alteration minerals within the GT3A, GT2A, and GT1A cores are similar, but their distribution varies. We map that variation in more detail and without complications of different people logging different sections of the core. While there are errors in our mapping as discussed in Section 4.2—it would be impossible to map the mineralogy of >1 billion pixels with 100% accuracy—the same methods are applied to every pixel, and errors are the same at all depths within each borehole and from one hole to the next.

**Figure 10:**
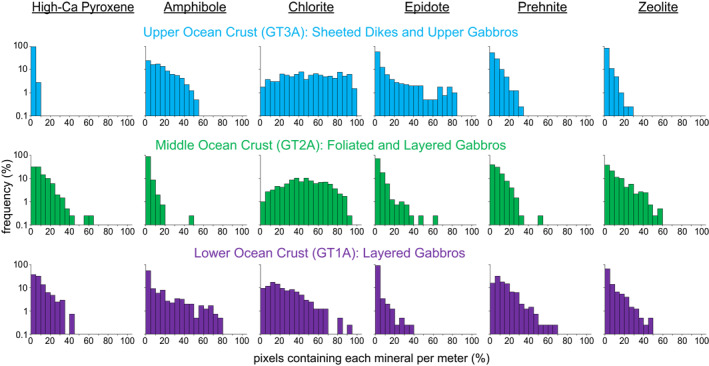
Histograms of the percentage of pixels in each meter of core containing key minerals in Holes GT3A (top), GT2A (middle) and GT1A (bottom). Histograms are calculated using bin sizes of 5%.

Overall, the prevalence of different alteration minerals in each borehole varies systematically (Table 3; Figures 7–10). As plagioclase is not mapped by imaging spectroscopy, we are only able to assess the extent of clinopyroxene replacement. We identify a spectral signature of high‐Ca pyroxene (augite/diopside) in <1% of the uppermost hole, GT3A. This does not mean pyroxene is absent, but it means that the progress of alteration is sufficiently extensive so as to obscure signatures of pristine pyroxene, and/or that fine grained clinopyroxene is underrepresented in the data set. In contrast, pyroxene occurrence is 9%–10% in the deeper holes, GT2A and GT1A (Table 3). Within Hole GT3A, 97% of 1‐m intervals had pyroxene identified in <5% of pixels, and no 1‐m intervals contained pyroxene in more than 10% of their area (Figure 10). In contrast, pyroxene was identified in >10% of pixels in 36% of 1‐m intervals within Hole GT2A and 31% in GT1A (Figure 10). Differences in igneous protolith or texture between the sheeted dikes of GT3A and the gabbros of GT2A and GT1A are not the control; pyroxene is similarly low in the lower gabbro sequence composing the bottom ∼170 m of Hole GT3A relative to the other cores (Figures 7, 8, 9). Thin section observations of background alteration of clinopyroxene within the gabbroic intervals in Hole GT3A indicate they are extensively pseudomorphed by amphiboles, whereas in Holes GT1A and GT2A clinopyroxene in areas of background alteration exhibit incipient alteration. Pyroxene abundance is therefore likely somewhat underestimated with VSWIR spectroscopy in all cores, especially Holes GT1A and GT2A, due to minor alteration of the pyroxenes and the surrounding minerals overwhelming the pyroxene signature and/or distorting the pyroxene structure sufficiently to lose the characteristic Fe^2+^ electronic transitions. Nevertheless, thin section petrography largely confirms the observed VSWIR trends by hole. The base of the sheeted dike complex and dike/gabbro transition zone is commonly assumed to be the locus of intensive hydrothermal exchange above the high level melt lens imaged at mid‐ocean ridges (e.g., Alt, 1995); our results are consistent with this interval experiencing pervasive alteration.

Pixel non‐detections (no pyroxenes nor the eight alteration minerals mapped in this paper) compose 44% of Hole GT1A and 29% and 28% of Holes GT2A and GT3A, respectively. These are likely areas of primary igneous plagioclase, which are transparent at the wavelengths measured, or locations with fine‐grained disseminated oxides that darken and can mask other spectral signatures (e.g., Morris et al., 1985). The close juxtaposition of such areas with regions of abundant clinopyroxene, particularly in Holes GT1A and GT2A (see File S2 and S3) further suggests that some sections of the cores experienced less hydrothermal alteration and that any alteration that did occur did not produce significant volumes of hydrated minerals, sulfates, or carbonates. In Hole GT3A, the pattern is less obvious, likely due to how infrequently pyroxenes are detected with spectroscopy. That the stratigraphically deepest hole (GT1A) has the most non‐detections makes sense if this material is fresher gabbro because the distributions of the alteration mineral groups that are mapped (Figure 9) show sections tens of meters long with less alteration between intensely altered zones.

Secondary minerals also exhibit different distributions in each borehole (Figures 7, 8, 9, 10; Table 3). In the sheeted dikes and uppermost gabbros of Hole GT3A, chlorite (51%), epidote (12%), and amphibole (15%) are the dominant secondary minerals, with zones of pervasive epidote and chlorite (Figures 7, 10). Some prehnite is present (6%), while zeolite alteration is relatively low (3%). Although still rare, zeolites are more abundant at depths >250 m within the Hole GT3A gabbros than in the sheeted dikes (Figure 7). At intermediate depth in the ocean crust, chlorite occurs in 46% of the foliated and layered gabbros of Hole GT2A, of similar overall occurrence percent to Hole GT3A, and Hole GT2A contains most spatially extensive zeolite (12%). Hole GT2A also has moderate amounts of prehnite (8% occurrence) and epidote (4% occurrence), but amphibole (3% occurrence) is rare relative to the other boreholes. Prehnite (14% occurrence) is more common in the deepest ocean crustal rocks, Hole GT1A. Chlorite (22% occurrence) is also abundant, although is notably less abundant than in the higher level boreholes. Zeolite (6% occurrence) is intermediate between Holes GT3A and GT2A, amphibole is similar to GT3A (13% occurrence), and epidote (2% occurrence) is low.

Although there are zones of pervasive amphibole alteration deeper, amphibole is rare in the shallowest 100 m of the Hole GT1A gabbros. Consistent with spectral interpretations, amphibole abundance is low in the uppermost 100 m based on thin section observations. In thin section, amphibole typically occurs as very fine grained laths intergrown with chlorite where this assemblage is replacing clinopyroxene (e.g., GT1A 27Z‐1 13–16 cm and GT1A 38Z‐3 21–24 cm; when present in thin section, imaging spectroscopy also identifies small spatial occurrences of amphibole in the corresponding billets). Amphibole is significantly more dominant within and surrounding fault zones, where amphibole is sometimes pervasive throughout the matrix and hydration is highest (Figure 9; see Crotteau et al., this issue, for a discussion of the hydration), discussed in more detail in Section 5.2.

A key question for investigation of any ophiolite is the extent to which mid‐ocean ridge hydrothermal exchanges and mineralogical changes are overprinted by later geological events that occurred since the emplacement of the ophiolite on the Arabian continental margin, including ongoing modern processes. Our results concur with core observations and suggest that lower temperature surficial weathering only affects the upper few 10's of meters at most. Kaolinite/montmorillonite and calcite show spikes in concentration at the uppermost portions of each borehole (Figures 7, 8, 9). Spatially extensive kaolinite/montmorillonite is present in the top 10–20 m of Holes GT3A and GT2A. In Hole GT1A, kaolinite/montmorillonite is observed to ∼50 m below the surface, with a second spike in occurrence within in a fault zone (Figure 9). In all boreholes, calcite typically extends deeper to ∼50 m, suggesting that the uppermost ∼10–50 m of each borehole has been weakly modified by modern surface weathering.

Gypsum often forms at low temperatures but does not exhibit a spike in occurrence near the surface as kaolinite/montmorillonite and calcite do (Figures 7, 8, 9). Rather, the rare occurrences deeper of petrographically late‐stage gypsum, particularly in GT1A, may therefore be locations of original gypsum precipitation or minor localized hydration, alteration or remobilization of anhydrite found during the core description (Kelemen et al., 2020).

### 5.2 Insights From Micro‐Imaging Spectroscopy of Hydrothermal Alteration in the Samail Ophiolite and Oceanic Crust

Infrared micro‐imaging spectroscopy identifies the same minerals as the core description teams (Kelemen et al., 2020) but with different distributions in some cases, particularly for prehnite, which we find in higher abundance, and amphibole. Consistent with the core description results, alteration is highly variable within each borehole, and there are often not clear trends of increasing or decreasing occurrence of particular minerals downhole, with a few exceptions. Comparing borehole to borehole, we find that the sheeted dikes and dike‐gabbro transition of Hole GT3A underwent pervasive greenschist facies alteration. Deeper alteration in the foliated and layered gabbros (Holes GT2A and GT1A) was concentrated in intervals of intense alteration between less altered gabbros. A direct comparison of occurrences of minerals here versus the core description is difficult because the core description teams logged different alteration types (background, halos, patches, and deformation related) separately whereas we assess all alteration together regardless of type/setting. However, the imaging spectroscopy data set provides some new insights beyond that observed by the core description teams and prior ocean drilling expeditions, particularly in identifying amphibole throughout and adjacent to major fault zones of the lower oceanic crust.

To date, scientific ocean drilling has only penetrated into the gabbros beneath the sheeted dikes in intact crust in Hole 1256D (e.g., Teagle et al., 2006, 2012; Wilson et al., 2006), although there has been drilling of gabbros in tectonic windows (e.g., Hess Deep: Gillis et al., 2014) and on slow spreading ridges (e.g., Hole 1309D, Exp. 304/305, Atlantis Massif—Blackman et al., 2011; Hole 735B, Southwest Indian Ridge—Dick et al., 2000). In Hole 1256D there is a step change in alteration mineralogy and temperatures from low temperature saponite‐rich alteration in the lavas to greenschist facies chlorite‐ and amphibole‐bearing alteration assemblages in the sheeted dikes and uppermost gabbros (Alt et al., 2010; Teagle et al., 2006, 2012; Wilson et al., 2006). Our results for the sheeted dikes in Hole GT3A are similar, with pervasive alteration, often greenschist facies, and common chlorite and amphibole (Figures 7, 8, 9, 10, Table 3). However, we also identify epidote in total nearly as frequently as amphibole in Hole GT3A (Table 3). The generally pervasive alteration in the sheeted dike complex that we and the OmanDP core description teams (Kelemen et al., 2020) identify with an assemblage of albite + chlorite + epidote + quartz ± prehnite is consistent with field based studies of the Semail ophiolite (Nehlig et al., 1994). However, the histograms in Figure 10 suggest that epidote occurrences in the Samail ophiolite tend to be localized, with epidote being more pervasive in those localized regions, in contrast to amphibole, which is present in small areas throughout a higher percentage of the core. In addition to the alteration of the sheeted dikes, we observe that the hydrothermal system extends into the gabbroic intervals of the dike‐gabbro transition in Hole GT3A with little change in the assemblage or spatial context of the key alteration minerals (Figure 7), consistent with the hypothesis of Harris et al. (2017) that hydrothermal alteration extends into the uppermost gabbros of the ocean crust.

Deeper in the ocean crust, in Holes GT2A and GT1A, we observe less widespread alteration. However, there are clear zones of intense alteration and also an increasing prevalence of lower temperature hydrothermal secondary mineralogy (Figures 8, 9, 10). With more pyroxene detected via spectroscopy (Table 3, Figure 10), deeper sections of the oceanic crust experienced less widespread and/or pervasive hydrothermal alteration and more localized alteration, for example, in fault zones. The presence in GT1A of chlorite, decreasing in spatial occurrence from the middle to lower ocean crust, and amphibole indicate high temperatures of alteration (likely greenschist facies), similar to that of Hole GT3A. Minerals formed through lower temperature hydrothermal alteration including prehnite and zeolite are substantially more common in Holes GT1A and GT2A. We observe, at the scale of borehole averages, a trend of increasing prehnite and decreasing epidote with depth (Table 3) and increasing occurrences of zeolite and prehnite with depth. Zeolite‐facies alteration, which is lower temperature than prehnite (e.g., Neuhoff & Bird, 2001), is most frequent in Hole GT2A.

**Figure 11:**
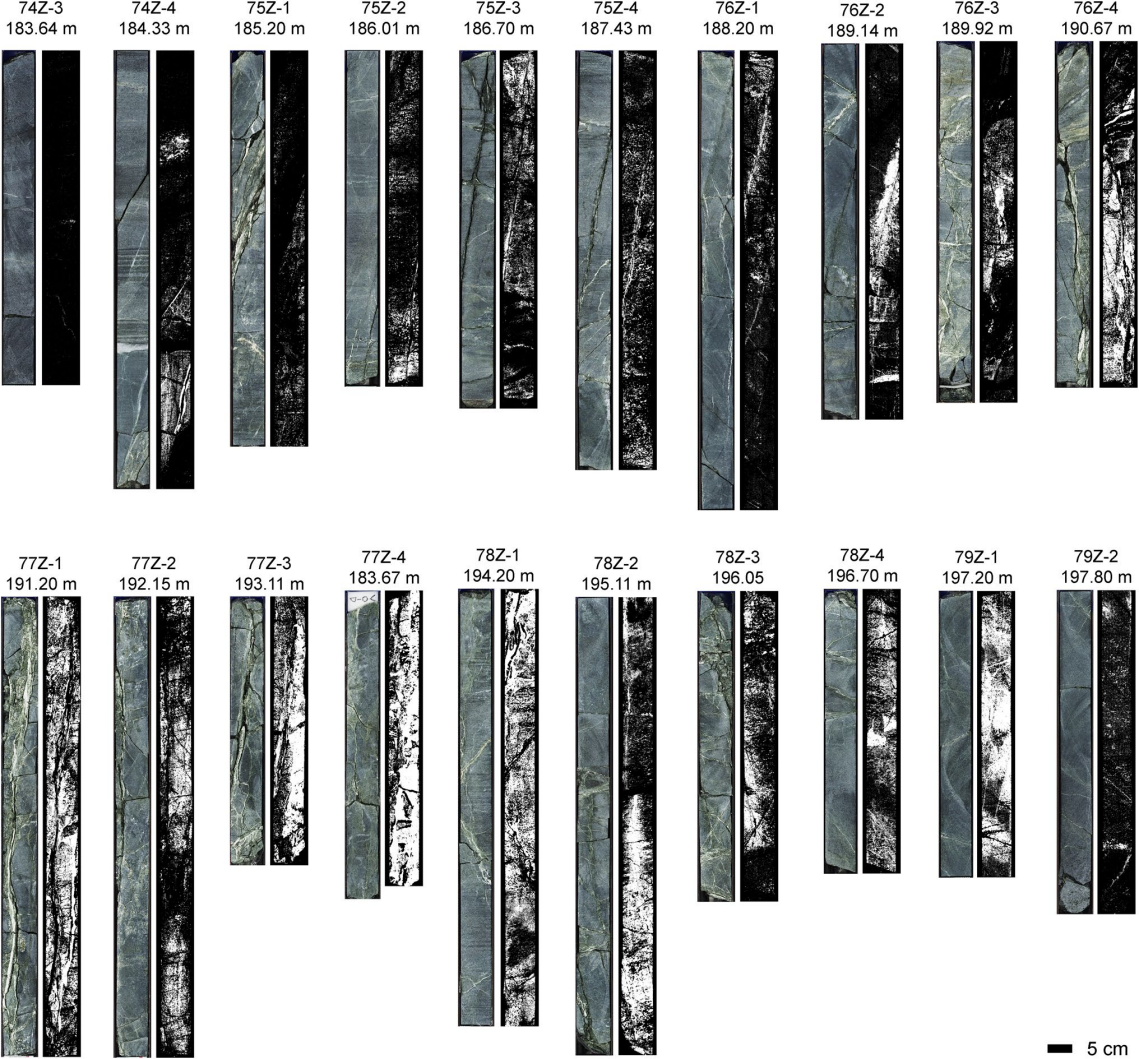
Distribution of amphibole within a major fault zone in Hole GT1A, labeled with core section and depth of the top of each section. Left panels are color scans from the multi‐sensor core logger on the Chikyu (Kelemen et al., 2020), and right grayscale panels show the depth of an absorption feature at ∼2.39 μm due to Mg‐OH in pixels where amphibole is present and is a proxy for amphibole abundance, though the depth can also have textural controls.

The high prevalence of amphibole in most major fault zones of the layered gabbros in the lower oceanic crust (Hole GT1A) is interesting (Figures 9 and 11). Other than an increase in chlorite at ∼80–90 m in Hole GT1A, the two major fault zones in the upper 150 m do not show clear depth‐dependent patterns with mineralogy (Figure 9). Below 150 m, fault zones are generally the most hydrated sections of Hole GT1A (Crotteau et al., this issue) and contain widespread amphibole, along with chlorite + prehnite + zeolite (Figures 9 and 11). Amphibole was identified during the core description but was likely underestimated, and the prominence of amphibole in fault zones compared with the surrounding rock is clearer in spectroscopy than downhole plots of mineral abundance based on core description alone (Kelemen et al., 2020). This clearly demonstrates the value of the imaging spectroscopy data set in identifying key areas of interest within the cores. While most XRD samples in fault zones targeted veins, a few included the matrix and validate our identification of an amphibole mineral (e.g., GT1A 77Z‐1 22–23 cm, labeled sample 2 in Figure 6, where actinolite was found; amphibole was also observed by both imaging spectroscopy and XRD in the same fault zone in sample GT1A 78Z‐4 24–25 cm). Thin section GT1A 77Z‐4 59–62 cm shows a pervasive amphibole matrix within this fault zone interval, but in addition to the mineralogy the thin section also displays highly variable textures and grain sizes that are finer than the resolution of the spectroscopy data set (Figure 4). Future work will determine precise amphibole mineralogy and assemblages, but actinolite is most likely since 11 of 17 XRD samples taken from the lower 250 m of Hole GT1A containing an amphibole mineral have actinolite. We note that there is a gap in the spectroscopy literature and libraries for distinguishing actinolite and hornblende across their full solid solutions in this wavelength range, and future work might improve this phase discrimination. Although we cannot yet constrain temperatures of amphibole formation, chlorite from surficial outcrops of deep crustal fault zones in Oman formed at 300°C–350°C (Zihlmann et al., 2018). Other minerals increase in occurrence in some but not all fault zones relative to the surrounding rock; zeolite is often elevated, occurrences of epidote are higher in fault zones at depths >250 m, and there are a few spikes in prehnite. The occurrence of lower temperature minerals suggests that fluid flow continued during cooling, especially concentrated across fault zones as temperatures decreased through epidote, prehnite, then zeolite‐facies alteration. As has been suggested from a study of epidote veins in the Samail ophiolite (Bieseler et al., 2018) and consistent with the OmanDP core description (Kelemen et al., 2020), our results highlight the importance of continued deep, off‐axis, low temperature fluid circulation that numerical models of fluid circulation in the ocean crust must take into account.

The first set of results from the OmanDP imaging spectroscopy data set with more than 1 billion measurements of mineralogy provides new insights into the hydrothermal alteration of the ocean crust, yet we have only scratched the surface of the data. Avenues for future work include quantification of mineral abundances with imaging spectroscopy and utilizing the data set to extrapolate ongoing geochemical measurements (e.g., isotopic and trace element) to the larger core. Petrography, electron microprobe or scanning electron microscopy measurements, and other traditional analytical techniques provide critical information but are not feasible on an entire length of drill core. Imaging spectroscopy of the OmanDP core fills gaps that petrology could never reach and moreover finds new patterns in the alteration and mineralogy of the ocean crust.

## 6 Conclusions

The processes through which the ocean crust forms, cools, and alters are not completely understood, in large part because of challenges accessing the crust deep below the ocean floor. The ICDP Oman Drilling Project recovered 3.2 km of core from the oceanic crust and upper mantle of the Samail Ophiolite, Oman, with near 100% recovery. We used micro‐imaging spectroscopy of 1.2 km of this core to systematically acquire more than 1 billion measurements of mineralogy of the sheeted dikes and dike‐gabbro transition in the upper oceanic crust (Hole GT3A), the foliated to layered gabbros at intermediate depth in the oceanic crust (Hole GT2A), and the layered gabbros with major fault zones in the lower oceanic crust (Hole GT1A). We characterized the mineralogy of nine key mineral groups formed through primary igneous processes (clinopyroxene) and hydrothermal alteration and low temperature weathering (amphibole, chlorite, epidote, gypsum, prehnite, zeolites, kaolinite/montmorillonite, and calcite) and validated the detections with thin section and XRD measurements obtained during the OmanDP core description (Kelemen et al., 2020). Except for infrequently occurring calcite, minerals identified with XRD are also identified in >80% of corresponding imaging spectroscopy data.

The downhole imaging spectroscopy trends show differences in pyroxene occurrence and alteration mineral distribution throughout the oceanic crust, with alteration of the sheeted dikes and dike‐gabbro transition dominated by greenschist facies assemblages of chlorite, amphibole, and prehnite but little lower temperature zeolite. More clinopyroxene remains in cores sampled from deeper in the oceanic crust, but zones of intense greenschist and lower temperature prehnite/zeolite facies alteration are present. In the deepest rock recovered by OmanDP, alteration is concentrated within and surrounding major fault zones, where we identify widespread amphibole. Our results suggest that fault zones are major conduits for fluid circulation in cooling and altering the lower oceanic crust.

## Supporting information

Supporting Information S1

Data Set S1

Data Set S2

Data Set S3

## Data Availability

All XRD measurements were published in Kelemen et al. (2020), and data are available from ICDP. Thin sections shown here were produced as part of Kelemen et al. (2020) and descriptions are available there. No new samples were analyzed in this work beyond the 1.2 km of OmanDP core, and samples can be requested through ICDP. The imaging spectroscopy data set are available through CaltechDATA: http://dx.doi.org/10.22002/D1.2009 (Greenberger, Ehlmann, & the Oman Drilling Project Science Party, 2021). Mineral occurrence maps (Files S2–S4) are part of the supplement of this study but are available through CaltechDATA due to their large size: http://dx.doi.org/10.22002/D1.2010.

## Appendix A

### A.1

**Table A1:** Formulas for Calculation of Spectral Parameters

Parameter	Center	Continuum	# Bands averaged	Attribution/rationale	Reference
Band depths (BD)^a^ and drops in reflectance (D)^b^					
BD1200	1.200	1.084, 1.300	3/3/3	Values >0.3 are often the edge of the core liner; optical effects make spectra of this edge very dark at longer SWIR wavelengths; the spectral features used to identify plastic materials elsewhere are often absent in the core liner	n/a
BD1390	1.39	1.352, 1.510	3/3/3	OH stretching overtone—Mg‐OH (wider continuum)	Bishop et al. (2002); Clark, King, et al. (1990)
BD1390_2	1.39	1.352, 1.427	3/3/3	OH stretching overtone—Mg‐OH (narrower continuum)	Bishop et al. (2002); Clark, King, et al. (1990)
BD1450broad	1.45	1.31, 1.67	5/3/3	OH stretching overtone in gypsum, zeolites, and other minerals with a bread feature	for example, Clark, King, et al., 1990; Cloutis et al., 2002; Greenberger et al. (2016)
BD1480	1.48	1.29, 1.6	3/3/3	OH stretching overtone in prehnite	Clark, King, et al. (1990)
BD1535	1.535	1.36, 1.77	3/3/3	OH stretching overtone in epidote (Fe^3+^‐rich)	Clark, King, et al. (1990); White et al. (2017)
BD1541	1.541	1.36, 1.77	3/3/3	OH stretching overtone in epidote	Clark, King, et al. (1990); White et al. (2017)
BD1550_max	Max of BD1535, BD1541, BD1553, BD1559, BD1565, and BD1571			OH stretching overtone in epidote; feature shifts with Al/Fe^3+^, and this is the maximum depth using the instrument^b^ andpasses	White et al. (2017)
BD1553	1.553	1.36, 1.77	3/3/3	OH stretching overtone in epidote	Clark, King, et al. (1990); White et al. (2017)
BD1559	1.559	1.36, 1.77	3/3/3	OH stretching overtone in epidote	Clark, King, et al. (1990); White et al. (2017)
BD1565	1.565	1.36, 1.77	3/3/3	OH stretching overtone in epidote (Al‐rich/clinozoisite)	Clark, King, et al. (1990); White et al. (2017)
BD1571	1.571	1.36, 1.77	3/3/3	OH stretching overtone in epidote	Clark, King, et al. (1990); White et al. (2017)
BD1715	1.715	1.685, 1.739	3/3/3	Organics (plastic, sponge, etc)	n/a
BD1760	1.76	1.655, 1.835	3/3/3	Gypsum; if present with 2.1 micron feature, can suggest organic	Cloutis et al. (2006); Hunt et al. (1971)
BD1760_narrow_field	1.751	1.727, 1.775	3/3/3	Gypsum	Cloutis et al. (2006); Hunt et al. (1971)
BD1900	1.93	1.825, 2.07	3/3/3	H‐O‐H combination	Clark, King, et al. (1990); Hunt and Ashley (1979)
BD2050pyx	2.05	1.67, 2.51	7/3/3	Fe^2+^ in high Ca pyroxene (e.g., augite, diopside)	Cloutis and Gaffey (1991)
BD2106	2.106	2.015, 2.21		If present with 1.7 micron feature, suggests organics/non‐rock	n/a
BD2150pyx	2.15	1.67, 2.51	7/3/3	Fe^2+^ in high Ca pyroxene (e.g., augite, diopside)	Cloutis and Gaffey (1991)
BD2120	2.12	2.094, 2.170	3/3/3	Feature in amphibole	Laukamp et al. (2012); Mustard (1992)
BD2200	2.206	2.134, 2.237	3/3/3	Al‐OH/Si‐OH	Aines and Rossman (1984); Anderson and Wickersheim (1964); Bishop et al. (2002); Clark, King, et al. (1990)
BD2210	2.217	2.185/2.245	3/3/3	S‐O bending overtone in gypsum, may also identify Al‐OH/Si‐OH	Clark, King, et al. (1990); Cloutis et al. (2006); Hunt et al. (1971)
BD2250_3	2.25	2.21, 2.27	3/3/3	Al/FeMg‐OH, continuum optimized for chlorite	Bishop et al. (2008); Clark, King, et al. (1990); Kokaly et al. (2017)
BD2250pyx	2.25	1.67, 2.51	7/3/3	Fe^2+^ in high Ca pyroxene (e.g., augite, diopside)	Cloutis and Gaffey (1991)
BD2255	2.255	2.10, 2.43	3/3/3	Likely Fe‐OH; continuum optimized for epidote	Clark, Gallagher, and Swayze (1990)
BD2300	2.3	2.12, 2.37	3/3/3	Fe/Mg‐OH combination band, will also identify carbonates	Clark, Gallagher, and Swayze (1990)
BD2300_carb	2.3	2.16, 2.34	3/3/3	C‐O feature in magnesite	Gaffey (1985, 1986, 1987); Hunt and Salisbury (1971)
BD2304	2.304	2.27, 2.358	3/3/3	Mg‐OH combination band in amphibole, will also identify other Mg‐bearing hydrated minerals and carbonates	Laukamp et al. (2012); Mustard (1992)
BD2310	2.31	2.27, 2.358	3/3/3	Mg‐OH combination band in amphibole, will also identify other Mg‐bearing hydrated minerals and carbonates	Laukamp et al. (2012); Mustard (1992)
BD2316	2.316	2.27, 2.358	3/3/3	Mg‐OH combination band in amphibole, will also identify other Mg‐bearing hydrated minerals and carbonates	Laukamp et al. (2012); Mustard (1992)
BD2322	2.322	2.27, 2.358	3/3/3	Mg‐OH combination band in amphibole, will also identify other Mg‐bearing hydrated minerals and carbonates	Laukamp et al. (2012); Mustard (1992)
BD2328	2.328	2.27, 2.358	3/3/3	Mg‐OH combination band in amphibole, will also identify other Mg‐bearing hydrated minerals and carbonates	Laukamp et al. (2012); Mustard (1992)
BD2310_amph	Max of BD2304, BD2310, BD2316, BD2322, and BD2328			Max band depth of ∼2.31 micron feature in amphibole due to solid solution chemistry	Laukamp et al. (2012); Mustard (1992)
BD2320	2.318	2.12, 2.37	3/3/3	C‐O in dolomite, will also identify Mg‐OH	Gaffey (1985, 1986, 1987); Hunt and Salisbury (1971)
BD2330_chlorite	2.33	2.16, 2.42	3/3/3	Mg‐OH	Bishop et al. (2008)
BD2340	2.34	2.18, 2.39	3/3/3	C‐O in calcite, will also identify Mg‐OH	Gaffey (1985, 1986, 1987); Hunt and Salisbury (1971)
BD2350pyx	2.35	1.67, 2.51	7/3/3	Fe^2+^ in high Ca pyroxene (e.g., augite, diopside)	Cloutis and Gaffey (1991)
BD2382	2.382	2.352, 2.442	3/3/3	Metal ‐OH combination (higher Mg#) in amphibole	Laukamp et al. (2012); Mustard (1992)
BD2388	2.388	2.352, 2.442	3/3/3	Metal‐OH combination in amphibole	Laukamp et al. (2012); Mustard (1992)
BD2394	2.394	2.352, 2.442	3/3/3	Metal‐OH combination in amphibole	Laukamp et al. (2012); Mustard (1992)
BD2400	2.4	2.352, 2.442	3/3/3	Metal‐OH combination in amphibole	Laukamp et al. (2012); Mustard (1992)
BD2400_AmphEp	2.4	2.38, 2.418	1/1/1	Metal‐OH combination in amphibole; optimized for subtle feature superimposed on epidote absorption	Laukamp et al. (2012); Mustard (1992)
BD2406	2.406	2.352, 2.442	3/3/3	Metal ‐OH combination (lower Mg#) in amphibole	Laukamp et al. (2012); Mustard (1992)
BD2390_amph	Max of BD2382, BD2388, BD2394, BD2400, BD2406			Max band depth of ∼2.39 micron feature in amphibole, accounting for shifts in minimum wavelength due to solid solution chemistry	Laukamp et al. (2012); Mustard (1992)
BD2400pyx	2.4	1.67, 2.51	7/3/3	Fe^2+^ in high Ca pyroxene (e.g., augite, diopside)	Cloutis and Gaffey (1991)
D2500	2.47	2.4	3/3	Drop in reflectance for carbonates toward 2.5 microns	Gaffey (1985, 1986, 1987); Hunt and Salisbury (1971)

Ratios	Numerator	Denominator	Averaging		
R1440/R1490	1.44	1.49	1/1	Distinguish gypsum from certain other sulfates	Greenberger et al. (2016)
R1457/R1490	1.457	1.480	1/1	Value is >1 for prehnite	Clark, King, et al. (1990)
RedSlope_SWIR	1.819	1.155	3/3	Often Fe^2+^ in minerals such as chlorite	Greenberger, Mustard, Cloutis, et al. (2015)
R2370/R2340	2.37	2.34	1/1	Value is >1 for carbonate	n/a

^a^ Band depth calculations: BD#=1‐R/Rc, where R is the average reflectance at the wavelength of interest and Rc is the value of a straight line continuum drawn between the given points (Clark & Roush, 1984). For each continuum endpoint, a median value is used with the number of adjacent bands given in the averaging column. The numbers in the averaging column is in the order center wavelength, shorter wavelength continuum, longer wavelength continuum. All wavelengths are in μm.

^b^ D# use the same formula except that *R*_c_ is the value of the continuum tie point on one side of the absorption feature and is typically used toward the edges of the wavelength range of the sensor where data are not available on one side of an absorption feature where the reflectance returns to the continuum.

**Table A2:** Formulas for Calculation of Mineral Indicators and Rock Mask Used to Exclude Non‐Rock Materials

Mineral	Chemical formula	Spectral feature(s) present	Spectral feature(s), mineral(s) absent	Key value for display	
Amphibole	NaCa_2_(Mg,Fe,Al)_5_(Al,Si)_8_O_22_(OH)_2_ (other cation substitutions common)	BD2310_amph > 0.018	n/a	BD2390_amph	
		BD2390_amph > 0.005			
		R2369_R2380 > 1.002			
		R2410_R2395 > 1.002			
Amphibole (with epidote)	(same as above)	Epidote	n/a	BD2400_AmphEp	
		BD2400_AmphEp > 0.002			
Calcite	CaCO_3_	BD2340 > 0.02	Chlorite	BD2340	
		BD2340 > BD2320	Epidote		
		BD2340 > BD2300_carb	Amphibole		
		D2500 > 0.02	Prehnite		
		R2370/R2340 > 1.08			
Chlorite	(Mg,Fe^2+^)_5_Al(Si_3_Al)O_10_(OH)_8_	BD2330_chlorite > 0.01	Amphibole	Maximum of BD1390 and BD1390_2	
		BD2250_3 > 0.003			
		BD1900 > 0.005			
		RedSlopeSWIR > 1			
		(BD1390 or BD1390_2) > 0.003			
Epidote/clinozoisite	Ca_2_(Fe^3+^,Al)_3_(SiO_4_)_3_(OH)	BD1550_max > 0.015	BD1760 < 0.03	BD1550_max	
		BD2255 > 0.01			
		BD2340 > 0.02			
		BD155_max_narrow > 0.01			
Gypsum	CaSO_4_·2H_2_O	BD1450broad > 0.02	n/a	BD1760	
		BD2210 > 0.005			
		BD1760 > 0.005			
		R1440/R1490 < 1			
		BD1760_narrow_field			
Kaolinite/montmorillonite	Al_2_Si_2_O_5_(OH)_4_	BD2200_kln > 0.02	Gypsum	BD2200_kln	
	(Na,Ca)_0,3_(Al,Mg)_2_Si_4_O_10_(OH)_2_•n(H_2_O)	BD2180_kln > 0.02			
		Kaolinite_slope > 1			
Prehnite	Ca_2_Al_2_Si_3_O_10_(OH)_2_	BD1480 > 0.005	n/a	BD1480	
		BD2340 > 0.005			
		R1457/R1480 > 1			
Pyroxene (high Ca)	(Ca,Mg,Fe)_2_Si_2_O_6_	BD2050pyx > 0.005	BD1450broad < 0.02	BD2250pyx
		Bd2150pyx > 0.01		
		BD2250pyx > 0.005		
		Bd2350pyx > 0.005		
		BD2400pyx > 0.005		
Zeolite group	CaAl_2_Si_4_O_12_•4(H_2_O) (laumontite)	BD1450broad > 0.03	BD2200 < 0.02	BD1900
		BD1900 > 0.05	BD2300 < 0.03	
			BD2340 < 0.02	
Rock mask	n/a—used to mask non‐rock materials (Styrofoam, core liner, plastic, labels, dark areas on the margin of images)	(R1110 < 0.02) or (R2410 < 0.02 and R1110 < 0.035)	n/a	n/a	
		–OR–			
		(BD1760 > 0.01 and BD2106 > 0.07) or (BD1715 > 0.05)			
		–OR–			
		BD1200 > 0.3			
