# Supplementary material for: Hydrothermal Alteration of the Ocean Crust and Patterns in Mineralization With Depth as Measured by Micro‐Imaging Infrared Spectroscopy
Source: J Geophys Res Solid Earth. 2021 Aug 24;126(8):e2021JB021976. doi: 10.1029/2021JB021976 (PMC8459238; doi:10.1029/2021JB021976)
Supplement: Supplementary file 1 — Supporting Information S1 [file JGRB-126-e2021JB021976-s002.pdf]

Journal of Geophysical Research – Solid Earth

Supporting Information for

Hydrothermal Alteration of the Ocean Crust and Patterns in Mineralization with Depth as Measured by Micro-Imaging Infrared Spectroscopy

Rebecca N. Greenberger[1], Michelle Harris[2], Bethany L. Ehlmann[1], Molly Crotteau[1], Peter B. Kelemen[3], Craig E. Manning[4], Damon A. H. Teagle[5], and the Oman Drilling Project Science Team

[1]Division of Geological and Planetary Sciences, California Institute of Technology, 1200 E. California Blvd., Pasadena, CA, 91125, USA

[2]School of Geography, Earth and Environmental Sciences, Plymouth University, Plymouth, PL4 8AA, UK

[3]Department of Earth & Environmental Sciences, Columbia University, Lamont–Doherty Earth Observatory, Palisades, NY 10964, USA

[4]Department of Earth, Planetary, and Space Sciences, University of California, Los Angeles, CA 90095, USA

[5]School of Ocean and Earth Science, National Oceanography Centre Southampton, University of Southampton, European Way, Southampton, SO14 3ZH, UK

Corresponding author: Rebecca Greenberger (rgreenbe@caltech.edu)

Contents of this file

Text S1
Tables S1 and S2

Additional Supporting Information (Files uploaded separately)

Captions for Supplement S2
Captions for Supplement S3
Captions for Supplement S4

Introduction

The supplementary data for this paper include the images and coordinates for each spectrum shown in Figs. 2-3 (Table S1), a table of the whether imaging spectroscopy identifies minerals detected with XRD by sample (Table S2), and mineral maps of all core sections from Holes GT1A (S2), GT2A (S3), and GT3A (S4). Files S2-S4 are PDF's and are available through CaltechDATA owing to their large size.

Supplementary Files S2, S3, and S4 show the mineral maps of each core section. The albedo that is shown for context is the median reflectance at 1.589-1.601 μm. All grayscale mineral images are the values of a key parameter given in Table A2 where the conditions are met for that mineral to be detected, also given in Table A2. The grayscale images are stretched linearly between the following values:

- Clinopyroxene (CPX): 0-0.25
- Amphibole: 0-0.12
- Epidote: 0-0.04
- Prehnite: 0-0.35
- Chlorite: 0-0.25
- Zeolite: 0-0.6
- Carbonate: 0-0.6
- Gypsum: 0-0.35
- Kaolinite/montmorillonite: 0-0.2

For the color map showing all minerals, minerals/mineral groups are drawn in the order: clinopyroxene, calcite, calcite + kaolinite, kaolinite, chlorite, epidote, prehnite, chlorite + epidote, epidote + amphibole, chlorite + prehnite, epidote + prehnite, chlorite + prehnite + epidote, zeolite, amphibole, gypsum. More mixtures are present than are shown in this image, which displays a limited number of combinations for graphical simplicity. Due to the order in which the colors are assigned for different minerals, some appear underrepresented relative to the grayscale images. For example, where the spectral signature suggests partial, weak alteration of pyroxene to amphibole or chlorite, the color mineral map will show amphibole or chlorite, not pyroxene. The grayscale maps do show the full distribution of pyroxene. These files are PDF's, and the first page gives the legend and scale. See main text in the paper for caveats, uncertainties, and processing steps.

File S2. Mineral maps of all core sections from Hole GT1A. The legend and scale information are given on the cover page.

File S3. Mineral maps of all core sections from Hole GT2A. The legend and scale information are given on the cover page.

File S4. Mineral maps of all core sections from Hole GT3A. The legend and scale information are given on the cover page.

Note: Files S2-S4 are just under 1 GB each and therefore are available through the CaltechDATA repository: http://dx.doi.org/10.22002/D1.2010.

Table S1. Core sections and pixels of each spectrum shown in Figs. 2-3. All spectra are 5x5 pixel averages centered at the given pixel. See data availability statement in main text for link to image cubes.

Mineral	Hole	Core section	Pixel
Clinopyroxene	GT1A	49Z-4	(91, 3241)
	GT2A	98Z-4	(71, 2370)
	GT3A	135Z-2	(168, 2795)
Amphibole	GT1A	111Z-2	(193, 2839)
	GT2A	24Z-3	(97, 1607)
	GT3A	121Z-4	(54, 2034)
Chlorite	GT1A	82Z-3	(182, 464)
	GT2A	15Z-4	(77, 2788)
	GT3A	92Z-3	(77, 1920)
Chlorite + epidote	GT1A	151Z-2	(101, 1056)
	GT2A	15Z-4	(267, 3986)
	GT3A	35Z-3	(245, 1900)
Chlorite + epidote + prehnite	GT1A	28Z-4	(86, 434)
	GT2A	22Z-3	(124, 2042)
	GT3A	87Z-3	(57, 498)
Chlorite + prehnite	GT1A	49Z-4	(124, 3465)
	GT2A	37Z-4	(88, 1301)
	GT3A	49Z-4	(65, 3141)
Epidote + amphibole	GT1A	111Z-2	(159, 2323)
	GT2A	33Z-4	(209, 1353)
	GT3A	108Z-1	(179, 1049)
Epidote	GT1A	111Z-2	(204, 2315)
	GT2A	24Z-3	(272, 2016)
	GT3A	89Z-2	(63, 2656)
Epidote + prehnite	GT1A	28Z-4	(232, 609)
	GT2A	21Z-3	(153, 1236)
	GT3A	115Z-4	(60, 2496)
Prehnite	GT1A	13Z-3	(178, 864)
	GT2A	21Z-3	(113, 1265)
	GT3A	71Z-3	(179, 1080)
Zeolite	GT1A	77Z-1	(242, 970)
	GT2A	12Z-1	(223, 1447)
	GT3A	58Z-2	(109, 965)
Gypsum	GT1A	77Z-1	(214, 1158)
	GT2A	18Z-4	(49, 729)
Calcite	GT1A	13Z-3	(271, 3334)
	GT2A	18Z-1	(101, 619)
	GT3A	10Z-2	(250, 3115)
Kaolinite	GT1A	13Z-3	(219, 3230)
	GT2A	2Z-1	(122, 2070)
	GT3A	2Z-2	(48, 1495)

Table S2. Validation of imaging spectroscopy with x-ray diffraction (XRD) measurements. ✓=identified in XRD and imaging spectroscopy; X=identified in XRD but not imaging spectroscopy. Other minerals are sometimes identified by XRD, but only the nine mapped with imaging spectroscopy are given in the table.

Hole	Section	Depth (cm)	Cpx	Amp	Chl	Ep/Czo	Prh	Zeo	Gp	Kln/Mnt	Cal
GT1A	7Z-3	40-46	✓				✓				
GT1A	13Z-1	24-25			✓			X			
GT1A	13Z-3	10-14					✓	✓			
GT1A	13Z-3	14-15	✓		✓		✓	✓			
GT1A	13Z-3	73-75	X					X		✓	✓
GT1A	14Z-1	73-74	X				✓	✓		X	
GT1A	20Z-1	63-64					✓	✓			
GT1A	24Z-3	76-77			✓		✓	✓			
GT1A	24Z-4	30-31					✓				
GT1A	25Z-3	0-2			✓		✓	✓			
GT1A	28Z-2	47-48	✓		✓		✓	✓			
GT1A	28Z-2	55-56			✓	✓		✓			
GT1A	28Z-2	63-63			✓		✓				
GT1A	28Z-4	3-4			✓	✓	✓	✓			
GT1A	28Z-4	19-20						✓			
GT1A	32Z-2	30-30	X		✓		✓				
GT1A	36Z-1	54-54	X		✓		✓	X			
GT1A	36Z-3	53-53	X		✓		✓				
GT1A	36Z-3	66-66	X	✓	✓		✓				
GT1A	37Z-2	30-30	✓		✓		✓	✓			
GT1A	39Z-1	28-28	✓		✓		✓				
GT1A	39Z-3	19-19	✓	✓	✓		✓				
GT1A	43Z-1	11-11			✓						
GT1A	49Z-2	77-77			✓			X			
GT1A	49Z-4	76-76	✓	✓	✓		✓				
GT1A	51Z-4	47-47									
GT1A	54Z-1	88-89					✓	✓			
GT1A	54Z-3	34-34			✓		✓	✓			
GT1A	55Z-3	7-7									
GT1A	55Z-3	25-25	✓				✓	✓			
GT1A	66Z-1	10-12	✓	✓	✓		✓				
GT1A	66Z-1	37-37		✓	✓		✓				
GT1A	66Z-4	75-76			✓			✓			
GT1A	67Z-1	12-12		✓			✓				
GT1A	67Z-2	21-22						✓	✓		
GT1A	68Z-3	42-43						✓			
GT1A	68Z-4	85-86			✓		✓	✓			
GT1A	76Z-3	45-46						✓			X
GT1A	77Z-1	21-22						✓	✓		
GT1A	77Z-1	22-23		✓				✓			
GT1A	78Z-4	24-25		✓				✓	X		
GT1A	80Z-2	46-47	✓				✓			X	
GT1A	81Z-1	4-5	✓	✓	✓			✓			
GT1A	82Z-3	9-10			✓						
GT1A	84Z-2	15-16	✓	✓			✓			X	
GT1A	85Z-1	24-24					✓				
GT1A	87Z-2	11-12	✓		✓			✓			

Hole	Section	Depth (cm)	Cpx	Amp	Chl	Ep/Czo	Prh	Zeo	Gp	Kln/Mnt	Cal
GT1A	87Z-3	45-46								✓	
GT1A	90Z-2	9-10						✓			
GT1A	91Z-2	11-12	✓								
GT1A	91Z-2	52-53	✓					✓			
GT1A	92Z-1	44-45	✓		✓		✓				
GT1A	102Z-4	8-9		✓	✓			✓			
GT1A	102Z-4	43-44	✓				✓	✓			
GT1A	104Z-2	64-66	✓	✓	✓						
GT1A	108Z-4	34-35		✓		✓					
GT1A	111Z-2	63-64		✓	✓						
GT1A	122Z-4	19-20						✓			
GT1A	131Z-4	29-30	✓		✓			✓			
GT1A	132Z-2	72-73	X		✓		✓	✓			
GT1A	148Z-2	27-29	✓		✓		✓	✓			
GT1A	151Z-2	21-24		✓	✓	✓	✓	✓			
GT1A	153Z-1	38-40	✓		✓		✓	✓			
GT1A	153Z-4	21-23	✓		✓		✓	✓			
GT2A	11Z-1	47-50	✓		✓			✓			
GT2A	11Z-1	62-63			✓	✓		✓			✓
GT2A	11Z-2	90-91			✓	✓		✓			✓
GT2A	12Z-1	30-32	X		✓			✓			
GT2A	13Z-2	0-2	X				✓	✓			✓
GT2A	13Z-2	43-54	✓		✓		✓	✓			
GT2A	13Z-2	52-53	✓		✓		✓	✓			
GT2A	13Z-4	9-10	✓		✓	✓	✓				✓
GT2A	14Z-3	9-10	X		✓			✓			
GT2A	15Z-4	54-57			✓			✓			
GT2A	15Z-4	74-77	✓		✓	✓		✓			
GT2A	15Z-4	79-89	✓		✓	✓		✓			
GT2A	16Z-2	15-16						✓			
GT2A	18Z-1	22-23		✓	✓		✓	✓			✓
GT2A	18Z-4	4-5			✓		X	✓	✓		
GT2A	19Z-1	73-75	✓				✓	✓			
GT2A	21Z-3	22-23				✓	✓				
GT2A	21Z-3	23-24				✓	✓	X			
GT2A	22Z-2	55-57	✓	✓	✓			✓			
GT2A	22Z-3	43-45	✓	✓	✓	✓	✓	✓			
GT2A	24Z-3	35-36	✓	✓		✓	✓				
GT2A	25Z-2	29-31	✓	✓				✓	✓		
GT2A	27Z-1	9-10		✓				✓			
GT2A	29Z-1	48-49	✓	✓	✓			✓			
GT2A	31Z-4	4-6	✓		✓		✓	✓			
GT2A	34Z-5	25-26	X		✓			✓			
GT2A	37Z-4	28-30	✓		✓		✓	✓			X
GT2A	39Z-4	54-55	✓				✓	✓			
GT2A	40Z-2	11-12	✓		✓			✓			
GT2A	41Z-4	31-32	✓	✓	✓		✓				
GT2A	50Z-3	17-18	✓		✓			✓			
GT2A	50Z-3	19-19	✓		✓			✓			
GT2A	55Z-3	18-19						✓	✓		✓
GT2A	61Z-1	16-17					✓	✓			
GT2A	64Z-2	76-77							✓		

Hole	Section	Depth (cm)	Cpx	Amp	Chl	Ep/Czo	Prh	Zeo	Gp	Kln/Mnt	Cal
GT2A	66Z-2	12-14							✓		✓
GT2A	70Z-1	3-4						✓			
GT2A	72Z-2	57-59						✓			
GT2A	73Z-4	17-18		✓	✓		✓				
GT2A	74Z-3	23-25			✓						
GT2A	81Z-4	54-54						X			
GT2A	81Z-4	54-54	✓	✓	✓		✓				
GT2A	84Z-3	47-48	✓		✓			✓			
GT2A	85Z-3	11-13					✓				
GT2A	85Z-3	56-58	✓				✓	✓			
GT2A	85Z-4	0-2	✓				✓	✓			
GT2A	86Z-2	56-63						✓			
GT2A	86Z-4	22-23			✓			✓	✓		
GT2A	87Z-4	51-52	✓				✓	✓			
GT2A	88Z-1	12-14					✓	✓			
GT2A	88Z-3	66-68						✓			
GT2A	89Z-3	17-18						✓	✓		
GT2A	89Z-4	58-60			✓		✓	✓	✓		
GT2A	90Z-1	44-45	✓		✓		✓	✓	✓		
GT2A	95Z-1	77-95	✓	✓	✓		✓	✓			
GT2A	95Z-4	44-46		✓	✓		✓				
GT2A	95Z-4	44-46			✓		✓				
GT2A	96Z-2	76-77	X				✓				
GT2A	98Z-1	19-20	✓		✓		✓				
GT2A	98Z-1	20-21					✓				
GT2A	98Z-4	56-57			✓		✓	✓			✓
GT2A	98Z-4	64-65	✓		✓			✓	X		
GT2A	100Z-3	16-18			✓						
GT2A	103Z-3	56-57			✓		✓				
GT2A	106Z-1	45-46	✓		✓		✓	✓			
GT2A	106Z-1	46-47	X		✓		✓	✓			
GT2A	110Z-1	26-27	✓		✓		✓	✓			
GT2A	110Z-3	34-34	✓		✓		✓	✓			
GT2A	113Z-1	41-42	✓	✓	✓						
GT2A	114Z-2	65-66	✓		✓		✓	✓			
GT2A	115Z-3	2-3					✓	✓			
GT2A	128Z-2	46-47	✓		✓		✓	✓			
GT2A	129Z-3	20-21	✓				✓	✓			
GT2A	135Z-1	2-4	✓		✓		✓	✓			
GT2A	136Z-1	52-54	✓	✓	✓						
GT2A	142Z-2	51-52	✓		✓						
GT3A	6Z-1	0-5		✓				✓			
GT3A	10Z-2	77-78			✓		✓	✓			X
GT3A	82-83	12Z-1		✓			✓	✓			
GT3A	14Z-1	64-65		✓				✓			
GT3A	29Z-2	43-44					✓	✓			
GT3A	34Z-1	11-12		✓	✓	✓					✓
GT3A	34Z-2	29-30				✓					X
GT3A	35Z-3	36-37		✓	✓	✓					
GT3A	39Z-3	37-38		✓		✓					
GT3A	39Z-4	48-49		✓	✓	✓					
GT3A	43Z-4	58-59		✓	✓	✓					

Hole	Section	Depth (cm)	Cpx	Amp	Chl	Ep/Czo	Prh	Zeo	Gp	Kln/Mnt	Cal
GT3A	44Z-4	25-27						✓			
GT3A	47Z-3	38-40			✓			X			
GT3A	49Z-4	65-66			✓		✓				
GT3A	55Z-2	37-38		✓				✓			
GT3A	56Z-3	10-11						✓			
GT3A	56Z-4	26-24						X			
GT3A	57Z-1	24-25						✓			
GT3A	58Z-2	16-17						✓			
GT3A	59Z-2	6-7		✓	✓	✓					
GT3A	59Z-2	27-28		✓	✓	✓					
GT3A	59Z-2	48-49		✓	✓	✓					
GT3A	59Z-2	59-60		✓	✓	✓					
GT3A	60Z-2	13-14					✓	✓			
GT3A	67Z-2	30-31					✓	✓			
GT3A	71Z-2	53-54					✓	✓			
GT3A	71Z-3	19-20					✓				
GT3A	80Z-2	9-10		✓			✓				X
GT3A	81Z-3	49-50		✓			✓				
GT3A	82Z-3	11-12		✓		✓					
GT3A	82Z-3	33-34		✓		✓					
GT3A	82Z-3	68-69		✓	✓						
GT3A	87Z-3	21-22		✓	✓						
GT3A	88Z-4	52-53		✓		✓					
GT3A	88Z-4	82-83				✓					
GT3A	89Z-2	31-32		✓	✓						
GT3A	89Z-2	52-53				✓					
GT3A	92Z-3	42-43			✓						
GT3A	94Z-1	30-31		✓	✓						
GT3A	100Z-4	34-35						✓			
GT3A	102Z-4	57-58		✓			✓	✓			
GT3A	106Z-1	48-52		✓			✓	✓			
GT3A	97Z-2	53-54		✓			✓	✓			
GT3A	107Z-4	5-10	X	✓	✓		X				
GT3A	108Z-1	20-21		✓	✓						
GT3A	108Z-1	28-29		✓		✓					
GT3A	113Z-1	20-21									
GT3A	113Z-1	20-21									
GT3A	115Z-4	54-55		✓		✓	✓				
GT3A	116Z-3	74-75		✓							
GT3A	116Z-4	25-56		✓				✓			
GT3A	116Z-4	43-44		✓							
GT3A	121Z-4	37-38		✓							
GT3A	122Z-1	28-38	✓	✓	✓						
GT3A	122Z-1	55-56		✓	✓			✓			
GT3A	122Z-1	57-58		✓	✓			✓			
GT3A	122Z-1	58-59		✓		✓		✓			
GT3A	123Z-4	29-30		✓	✓		✓	✓			
GT3A	131Z-4	12-13		✓							
GT3A	133Z-4	72-73		✓	✓			✓			
GT3A	135Z-2	15-17	✓	✓	✓			✓			
GT3A	135Z-3	8-9		✓	✓		✓				
GT3A	136Z-2	54-55					✓				

Hole	Section	Depth (cm)	Cpx	Amp	Chl	Ep/Czo	Prh	Zeo	Gp	Kln/Mnt	Cal
GT3A	141Z-3	23-24		✓		✓					
GT3A	141Z-3	45-46		✓				✓			✓
GT3A	142Z-2	58-60		✓	✓			✓			
GT3A	144Z-3	23-24				1					
GT3A	145Z-3	6-7		✓							
GT3A	147Z-1	72-73			✓	✓					
GT3A	151Z-4	69-70		✓			✓	✓			
GT3A	153Z-1	47-48		✓				✓			

Cpx=clinopyroxene; Amp=amphibole; Chl=chlorite; Ep/Czo=epidote/clinozoisite; Prh=prehnite; Zeo=zeolite; Gp=gypsum; Kln/Mnt=montmorillonite; Cal=calcite
