# Supplementary material for: Hydrothermal Alteration of the Ocean Crust and Patterns in Mineralization With Depth as Measured by Micro‐Imaging Infrared Spectroscopy
Source: J Geophys Res Solid Earth. 2021 Aug 24;126(8):e2021JB021976. doi: 10.1029/2021JB021976 (PMC8459238; doi:10.1029/2021JB021976)
Supplement: Supplementary file 3 — Data Set S2 [file JGRB-126-e2021JB021976-s001.pdf]

File S3 is just under 1 GB and is available at http://dx.doi.org/10.22002/D1.2010. This PDF contains the mineral maps from Hole GT2A.
